# Copy Number Amplification of DNA Damage Repair Pathways Potentiates Therapeutic Resistance in Cancer

**DOI:** 10.7150/thno.39341

**Published:** 2020-03-04

**Authors:** Zhiyuan Wu, Sihan Li, Xuemei Tang, Yue Wang, Weiwei Guo, Guojun Cao, Kun Chen, Min Zhang, Ming Guan, Da Yang

**Affiliations:** 1 Department of Laboratory Medicine, Huashan Hospital, Fudan University, Shanghai 200040, China.; 2 Center for Pharmacogenetics, Department of Pharmaceutical Sciences, University of Pittsburgh, PA 15261, USA.; 3 Central Laboratory, Huashan Hospital, Fudan University, Shanghai 200040, China.; 4 University of Pittsburgh Cancer Institute, University of Pittsburgh, Pittsburgh, PA 15261, USA.; 5 Department of Computational and Systems Biology, University of Pittsburgh, Pittsburgh, PA, 15261, USA.

**Keywords:** DNA damage repair, chemotherapy resistance, pharmacogenetics

## Abstract

**Rationale**: Loss of DNA damage repair (DDR) in the tumor is an established hallmark of sensitivity to DNA damaging agents such as chemotherapy. However, there has been scant investigation into gain-of-function alterations of DDR genes in cancer. This study aims to investigate to what extent copy number amplification of DDR genes occurs in cancer, and what are their impacts on tumor genome instability, patient prognosis and therapy outcome.

**Methods**: Retrospective analysis was performed on the clinical, genomics, and pharmacogenomics data from 10,489 tumors, matched peripheral blood samples, and 1,005 cancer cell lines. The key discoveries were verified by an independent patient cohort and experimental validations.

**Results**: This study revealed that 13 of the 80 core DDR genes were significantly amplified and overexpressed across the pan-cancer scale. Tumors harboring DDR gene amplification exhibited decreased global mutation load and mechanism-specific mutation signature scores, suggesting an increased DDR proficiency in the DDR amplified tumors. Clinically, patients with DDR gene amplification showed poor prognosis in multiple cancer types. The most frequent Nibrin (*NBN*) gene amplification in ovarian cancer tumors was observed in 15 out of 31 independent ovarian cancer patients. *NBN* overexpression in breast and ovarian cancer cells leads to BRCA1-dependent olaparib resistance by promoting the phosphorylation of ATM-S1981 and homology-dependent recombination efficiency. Finally, integration of the cancer pharmacogenomics database of 37 genome-instability targeting drugs across 505 cancer cell lines revealed significant correlations between DDR gene copy number amplification and DDR drug resistance, suggesting candidate targets for increasing patient treatment response.

**Principal Conclusions**: DDR gene amplification can lead to chemotherapy resistance and poor overall survival by augmenting DDR. These amplified DDR genes may serve as actionable clinical biomarkers for cancer management.

## Introduction

Tumors with DNA damage repair (DDR) deficiency demonstrate sensitivity to genome-instability targeting chemotherapies through “synthetic lethality” 1-3. DDR targeting agents have shown promising benefit in the precision medicine for cancer 4-8. Poly ADP ribose polymerase inhibitor (PARPi) is the most remarkable example of such DDR targeting agents, and has shown success in both *in vitro* studies and cancer patients 9,10. This led to the FDA's consecutive approval of olaparib (2014) 11, rucaparib (2016) 12, niraparib (2017) 13, and talazoparib (2018) 14, for the treatment of advanced ovarian cancer and metastatic breast cancer patients with germline *BRCA* mutation.

Conversely, the restoration of DDR pathway function (e.g., revival of homology-dependent recombination (HDR) by loss of *53BP1* 15 or *REV7* 16 in *BRCA* mutated cancer patients) introduces resistance to those DDR targeting agents. Despite the previously well-established connections between DDR loss-of-function and cancer development and treatment 17-19, how frequently the gain-of-function alterations in DDR pathways occur in cancer, and to what extent they affect the DNA damage repair clinical outcome and even drug response remain elusive. In this study, we aimed to characterize the landscape of copy number amplification across nine DDR pathways in cancer by integrating the multi-dimensional genomic data from primary cancer samples and cancer cell lines across 32 cancer types. By further integrating the DDR gene data with tumor mutation burden, mutation signature, clinical treatment information and cancer cell line pharmacogenomics data, we sought to determine the DDR gene amplifications' impacts on the tumor genome instability, patient prognosis and drug responses.

## Methods

### Characterization of DDR gene copy number amplification and overexpression across 32 cancer types

RNA-Seq gene expression, somatic mutation and somatic copy number alteration (SCNA) of 80 “core-list” from 276 “full-list” DNA Damage Repair (DDR) genes 20 in 10,489 primary tumors were obtained from the TCGA PanCancerAtlas 21 cohort consisting of tumor patients across 32 cancer types. The copy number segmentation data (SCNA score) were obtained from the Circular Binary Segmentation (CBS) algorithm 22, and the GISTIC (Genomic Identification of Significant Targets in Cancer) calls comprising -2 (deletion), -1 (loss), 0 (diploid), 1 (gain), and 2 (amplification) were made using GISTIC2.0 20. mRNA expressions data and copy number alterations of the 80 core DDR genes across 1,005 cancer cell lines were downloaded from the Genomics of Drug Sensitivity in Cancer (GDSC) database 23. Genes with over 5% of samples harboring GISTIC call = -2 or 2 in more than two cancer types were defined as recurrently copy number deleted or amplified. A pathway is labeled as significantly amplified in one sample if at least one gene in the pathway showed amplification in the sample with a false discovery rate (FDR) < 0.25 (see **Supplementary Methods**). Spearman's rank correlation coefficient was used to detect the correlation between gene expression and copy number alteration for each gene in the cell lines and patient samples respectively. Gene Set Enrichment Analysis (GSEA) 24 was performed to further interpret the association between DDR gene amplification and mRNA overexpression (see** Supplementary Methods**).

### Assessment of the relationship between tumor genome stability and DDR gene copy number amplification in the TCGA patient samples

The tumor genome stability information, including mutation burden and mutation signature scores for the PanCancerAtlas patients was obtained from The Cancer Genome Atlas (TCGA) database 21. Two-sample *t*-test was used to show the difference in mutation burden/mutation signature scores between samples containing copy number amplifications (GISTIC call =2) of a specific gene vs. the other samples. GSEA analysis was performed on the gene list ranked by the correlation between the gene copy number and mutation burden to determine whether DDR pathways are enriched in the top genes whose copy number gain/amplification could decrease genome instability.

### Chemotherapy and radiotherapy information of cancer patients and survival analysis

The raw clinical data of 10,237 TCGA patients across 33 cancer types were obtained from the Genomic Data Commons (GDC). Chemotherapy, radiotherapy and patient survival information were extracted from the raw TCGA clinical data as we previously reported 25 (see** Supplementary Methods**). Overall survival rates were estimated by Kaplan-Meier curves between patients with or without specific gene copy number amplification/gain (CNAmp; GISTIC calls = 1 and 2) versus others and compared in the specific cancer types using a Cox regression model stratified by the DDR gene SNCA score.

### Association analysis between DDR gene copy number alteration and cell line drug response

Drug response data of 37 genome-instability targeting drugs across 1,005 cancer cell lines were downloaded from the GDSC database 23 (see** Supplementary Methods**) and processed as in our previous report 25. Five hundred and five cell lines with multi-dimensional pharmacogenomics data available were retained for the following analysis. A logarithmic transformed half maximal inhibitory concentration (IC50) value was used to indicate the drug response in each cell line. Correlation between gene copy number alteration and treatment response to each drug was calculated by Spearman's rank correlation coefficient. The difference between drug responses in the cell lines bearing different DDR gene copy numbers was determined by Wilcoxon rank-sum test.

### Laboratory Methods

The copy number and protein expression of *NBN* were determined in formalin fixed paraffin embedded (FFPE) ovarian cancer/para-cancerous tissues from 31 serous epithelial ovarian cancer patients seen in the Department of Gynecological Surgery in the Obstetrics & Gynecology Hospital of Fudan University, by digital droplet PCR (ddPCR) and immunohistochemistry respectively. Written informed consent was obtained from all participants. This study obtained institutional review board approval for the characterization of these molecular features of tumor samples from each patient.

Human breast cancer cell line MCF-7 was a kind gift from Dr. Shilpa Sant. Human ovarian cancer cell lines OVCAR4 and SK-OV3 were purchased from Charles River Laboratories (Frederick, MD) and American Type Culture Collection (ATCC) (Manassas, VA). HA-NBN was stably overexpressed in the cell lines by lentiviral infection. *NBN*, *BRCA1*, or *ATM* was knocked down by siRNA in the cell lines. The cell lines were treated with olaparib (LC laboratories, #O-9201) or cisplatin (Selleckchem, #S1166) for drug response assay, or (S)-(+)-camptothecin (Sigma-Aldrich, #C9911) for double-strand break induction, before the cellular phenotype assays. Cell viability was determined by MTT assay using the CellTiter cell proliferation assay kit (Promega, #G4100), or clonogenic assay by crystal violet staining. Homology-dependent recombination efficacy was determined by RAD51 foci formation assay by immunofluorescence-based foci counting. The *in-vivo* tumor model was developed using nude mice by subcutaneous injection of SK-OV3 cancer cells with or without NBN overexpression. After administration of cisplatin, olaparib, or saline by intraperitoneal injection, the tumor drug response was assessed as tumor weight [drug treatment]/tumor weight [saline treatment]. Detailed information for laboratory experiments is provided in the **Supplementary Methods**.

## Results

### A systematic analysis revealed that DDR genes are significantly amplified and overexpressed in cancer patients

We focused on scrutinizing the copy number amplification alterations of 80 “core” DNA damage repair (DDR) genes 20 composing nine major DDR pathways, including Base Excision Repair (BER), Direct Repair (DR), Fanconi Anemia (FA), Homology-Dependent Recombination (HDR), Mismatch Repair (MMR), Non-homologous End Joining (NHEJ), Nucleotide Excision Repair (NER), Translesion Synthesis (TLS), Damage Sensors and Others. Consistent with previous studies 20, our mutation analysis did not identify recurrent gain-of-function point mutations in the DDR genes (data not shown).

**Figure 1:**
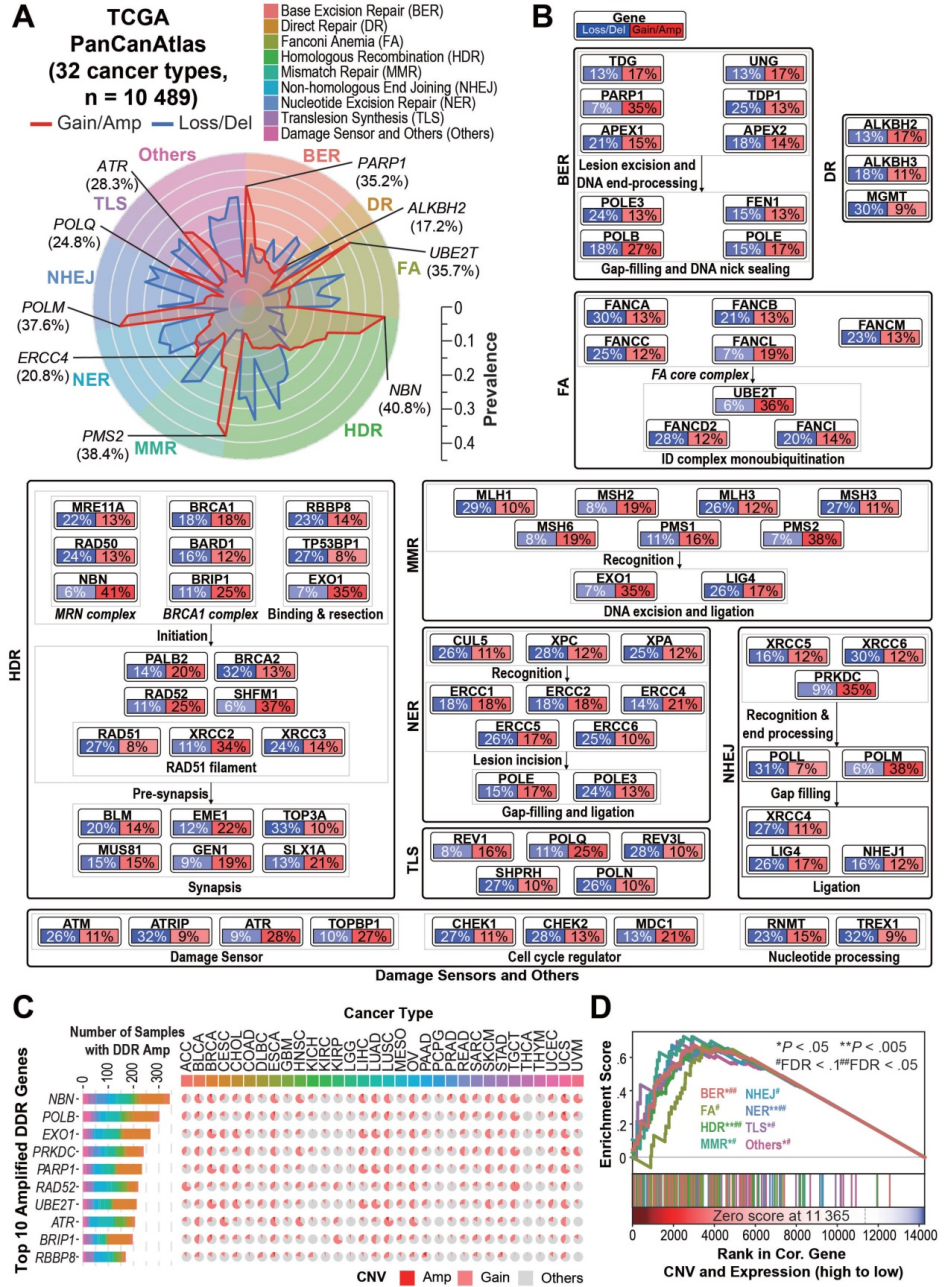
** Overview of DDR core gene amplification across pan-cancer. A**) Radar plot of the prevalence of copy number gain/amplification (red line) and loss/deletion (blue line) events in the 80 “core” DDR genes across nine DDR pathways among the 10,489 TCGA pan-cancer tumors. The most prevalent amplified gene in each DDR pathway is marked in italics with the pan-cancer prevalence. The prevalence of the copy number alteration events is indicated by the scale bar. **B**) Pan-cancer pathway mapper for the gene-level copy number alteration prevalence of 80 “core” genes across nine DDR pathways. **C**) The bar chart (left) indicates the total number of cancer patient samples showing amplification (GISTIC call = 2) in the top 10 amplified DDR genes, and the pie chart (right) shows the cancer-specific prevalence of gene amplification (red, GISTIC call = 2) and gain (pink, GISTIC call = 1) for the top 10 amplified DDR genes. For a complete list of TCGA cancer type abbreviations, please see https://gdc.cancer.gov/resources-tcga-users/tcga-code-tables/tcga-study-abbreviations. **D**) The Gene Set Enrichment Analysis (GSEA) of DDR pathways on genes ranked based on their correlation between expression and copy number alterations in the pan-cancer tumors.

Intriguingly, we observed recurrent DDR gene copy number amplifications/gains (CNAmps) among the 10,489 TCGA cancer samples across 32 tumor types (**Figure 1A**, **1B**, **Table S1**, and **Figure S1A**). Among the nine DDR pathways, pathways responsible for the double strand break (DSB) restoration (HDR and NHEJ), as well as the damage sensors (FDR = 0.13) were significantly amplified across the pan-cancer cohort (FDR < 0.25). The HDR pathway, as the most prevalently amplified DDR pathway (76.8% of pan-cancer), was significantly amplified over 18 cancer types including lung adenocarcinoma (LUAD, 464 [90.8%] of 511), rectum adenocarcinoma (READ, 142 [91.6%] of 155), ovarian serous cystadenocarcinoma (OV, 544 [96.8%] of 562), lower grade glioma (LGG, 307 [60.2%] of 510)/glioblastoma (GBM, 526 [92.1%] of 571) and breast invasive carcinoma (BRCA, 948 [88.6%] of 1070) (FDR < 0.25). We observed that cancer types sharing similar tissue origins or carcinogenic risk factors harbor similar DDR gene CNAmp patterns, such as seen in colon adenocarcinoma (COAD) and READ (r = 0.97, *P* = 6.2×10^-52^), head and neck squamous carcinoma (HNSC) and esophageal carcinoma (ESCA) (r = 0.83, *P* = 2.9×10^-21^), and liver hepatocellular carcinoma (LIHC) and cholangiocarcinoma (CHOL) (r = 0.83, *P* = 9.3×10^-22^) (**Figure S1B**).

On the individual gene level, 13 of the 80 core DDR genes showed significantly recurrent amplification among multiple cancer types (genes amplified in over 5% of samples in more than two cancer types, see **Supplementary Methods**). In contrast, only 3 DDR genes showed significantly recurrent deletion under the same criteria (**Table S2**). The most frequently amplified genes across the Pan-Cancer Atlas were *NBN* (n = 4,275, 40.8%), *EXO1* (n = 3,714, 35.4%), *PARP1* (n = 3,695, 35.2%), *PRKDC* (n = 3,650, 34.8%) and *POLB* (n = 2,794, 26.6%) (**Figure 1C**). All of the 13 recurrently amplified DDR genes exhibited significant overexpression in the amplified tumors (*P* < 10^-20^, **Table S2**). Gene Set Enrichment Analysis (GSEA) revealed that overexpression of all of the nine DDR pathways in the tumors is significantly driven by their CNAmp (FDR < 0.1) (**Figure 1D**). To further validate the DDR pathways' CNAmp and their overexpression in cancer, we investigated the DNA copy number and mRNA expression data of 1,005 cancer cell lines from the Genomics of Drug Sensitivity in Cancer (GDSC) database 23. The overexpression of all 13 of the recurrently amplified DDR genes was significantly driven by copy number amplification in the cancer cell lines (**Figure S1C** and **Tables S2** and **S3**).

### Tumors with DDR gene CNAmp exhibit decreased tumor genome instability and reduced mutational signature scores

**Figure 2:**
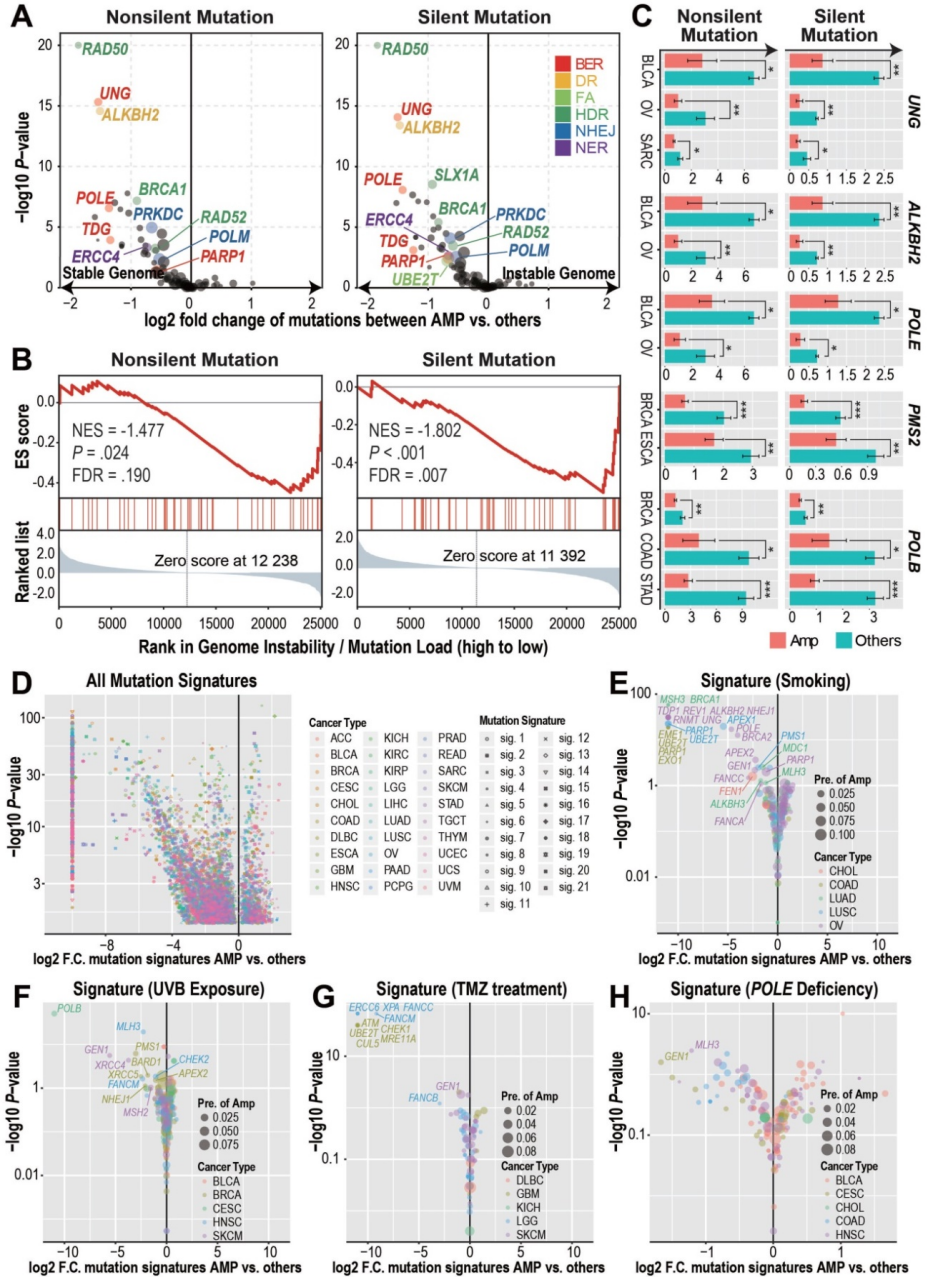
** DDR gene amplified tumors harbor reduced genome instability. A**) DDR gene amplification is associated with reduced mutation burdens (pan-cancers). Student's *t*-test results showed significant differences in both the silent and non-silent mutation burdens between tumors with or without individual DDR gene amplification. Log2-transformed fold changes of the mutation burden scores between the amplified samples versus others and their corresponding negative log10-transformed *t*-test *P*-values are shown in the x-axis and y-axis of the volcano plot. The prevalence of DDR gene amplification is indicated by the size of each circle. **B**) GSEA analysis revealed that BER pathway amplification is associated with reduced tumor mutation burden in TCGA ovarian cancers. The genes are ranked based on the mutation burden differences between ovarian cancer tumors with or without each gene's CNAmp. **C**) Significant cancer specific silent/non-silent mutation burden reduction in the tumors with individual DDR gene amplification (pink) compared to tumors without amplification (green) by Student's *t*-test. Error bars indicate mean ± SEM. *: *P* < 0.05, **: *P* < 0.005, ***: *P* < 0.0005. **D**) DDR gene amplification is significantly associated with reduced mutation signature scores of 21 different mechanisms (shapes) in each cancer type (colors) by Student's *t*-test. Log2-transformed fold change of each mutational signature score between samples with or without individual DDR gene amplification and its negative log10-transformed *t*-test *P*-value are shown in the x-axis and y-axis of the volcano plot respectively. **E-H**) Mechanism specific, such as smoking- (**E**), UVB exposure- (**F**), temozolomide treatment- (**G**), and *POLE* deficiency- (**H**) induced mutation signatures decreased in the DDR gene-amplified tumors in different cancer types. The size of each circle indicates cancer specific gene amplification prevalence in the pan-cancer samples and colors specify diverse cancer types.

With the observation of significantly recurrent overexpression and amplification of DDR genes in both primary tumors and cancer cell lines, we wondered if CNAmp and overexpression of DDR genes would increase the DDR function in tumor cells. In this regard, we investigated whether there was a difference in the mutation burdens 20 between the tumors with or without DDR gene CNAmp. This analysis revealed that tumors harboring CNAmp of 11 individual DDR genes (4 of which are recurrently amplified among multiple cancer types, *UBE2T*, *PARP1*, *PRKDC*, and *RAD52*) exhibited significantly reduced mutation burden versus those without CNAmp of these 11 DDR genes (**Figure 2A**), suggesting that the amplification of DDR genes might lead to an increased DDR function in those tumors. For example, the amplification of the BER pathway, including the genes *UNG*, *POLE*, *TDG*, and *PARP1*, is prominently correlated with genome stability in the OVs, as tumors with a stable genome were significantly enriched in the BER pathway gene amplified sample set (NES = 1.802, FDR = 0.007) (**Figure 2B**).

Cancer type-specific mutation burden analysis at the gene level further confirmed the association between DDR gene CNAmp and increased tumor genome stability (**Figure 2C** and **Figure S2A**). For instance, *POLE* is a gene involved in the BER pathway. Its loss-of-function mutations have been established to cause a hyper-mutator phenotype in multiple cancer types 26. In our study, we found that *POLE* amplified tumor samples exhibited 50% reduced mutation burden than tumors without *POLE* amplification in bladder urothelial carcinoma (BLCA) and OV (**Figure 2C**), suggesting that the gain-of-function *POLE* CNAmp may lead to an increased DDR function in cancer.

In addition to using the mutation burden as an indicator for global genome instability, we further compared the mechanistic specific mutation spectrum between the DDR gene amplified tumors and other tumor samples to determine if DDR gene CNAmp could alter the recurrent mutations accumulated under specific mutation sources and mutation mechanisms 27,28. When considering all the 21 previously defined mutation signatures 28, including smoking-, UVB exposure-, and *POLE* deficiency-induced mutation signatures, we observed that tumors with DDR gene amplification have a significantly lower incidence of the aforementioned DNA damage (**Figure 2D-H** and **Figure S2B**). For example, LUSCs, COADs, and OVs with *PARP1* amplification have a significant reduction in the smoking-induced mutation signature (LUSC, fold change < 10^-3^, *P* = 3.09×10^-23^; COAD, fold change < 10^-3^, *P* = 1.74×10^-19^; OV, fold change = 0.45, *P* = 0.01) (**Figure 2E**), which indicates a critical role for the error-free BER pathway genes in amending the smoking-induced DNA lesions 29. Tumors bearing amplification of FA pathway genes (*FANCB*, *FANCC* and *FANCM* in LGG and *UBE2T* in GBM) and HDR genes (*ATM*, *CHECK1*, *MRE11A* in GBM and *GEN1* in skin cutaneous melanoma [SKCM]) showed significantly reduced temozolomide-induced signature score (**Figure 2G**), indicating the critical role of DSB-associated recombinational repair in attenuating alkylating agent-induced genome lesions 30,31. These observations suggest that the CNAmp of DDR genes would restore the DDR function in tumor cells, thus alleviating the genome lesions and maintaining genome stability in the tumor.

### DDR gene CNAmp in the tumor is significantly correlated with poor cancer patient survival

**Figure 3:**
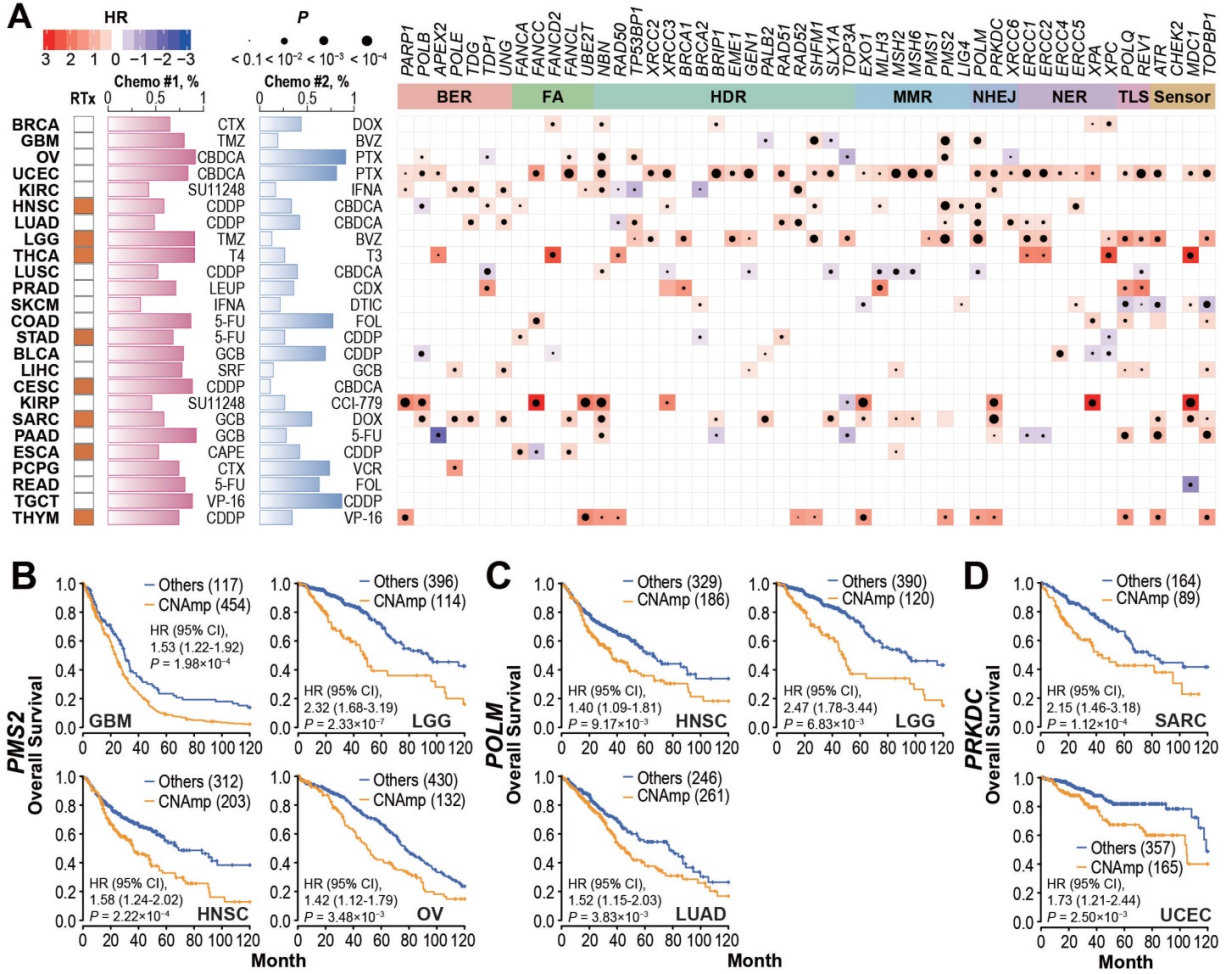
** DDR gene amplification in the tumor correlates with poor patient survival and drug resistance. A**) Tumor patients with specific DDR gene CNAmps (GISTIC calls = 1 and 2) showed poor overall survival compared to the rest of the patients by the Cox regression model. The radiotherapy and chemotherapy background of each cancer type is shown in the left panel. RTx: radiotherapy; Yellow: radiotherapy is commonly applied. Chemo #1, Chemo #2: the top 2 commonly used chemotherapy agents; percentage of patients that received each agent is indicated by the bar chart. CTX: Cyclophosphamide; DOX: Doxorubicin; TMZ: Temozolomide; BVZ: Bevacizumab; CBDCA: Carboplatin; PTX: Paclitaxel; SU11248: Sunitinib; IFNA: Interferon A; CDDP: Cisplatin; T4: Levothyroxine; T3: Liothyronine; LEUP: Leuprolide; CDX: Bicalutamide; DTIC: Dacarbazine; 5-FU: Fluorouracil; FOL: Leucovorin; GCB: Gemcitabine; SRF: Sorafenib; CCI-779: Temsirolimus; CAPE: Capecitabine; VCR: Vincristine; VP-16: Etoposide. Right panel: Heat map; blue and red represent negative and positive hazard ratios, respectively and *P*-value is denoted by the dot size. DDR genes with CNAmp in over 35% of pan-cancer patients are presented here. **B-D**) *PMS2* (**B**), *POLM* (**C**), and *PRKDC* (**D**) amplification is significantly associated with poor survival in multiple cancer types. Overall survival rates were estimated by Kaplan-Meier curves between patients with or without specific gene CNAmp and compared in the specific cancer types using a Cox regression model using the DDR gene SCNA score.

To investigate whether DDR gene amplification is clinically relevant, we analyzed the correlation between patient overall survival and DDR gene CNAmp in each cancer type. As shown in **Figure 3A** and **Figure S3A**, the CNAmp of core DDR genes exhibited a broad correlation with unfavorable survival of cancer patients. For example, *PMS2* is a critical component of the MutL alpha heterodimer for the initiation of mismatch repair 32, 33. Amplification of the *PMS2* gene is frequently found in glioblastoma multiforme (GBM, 454 [79.5%] of 571), lower grade glioma (LGG, 134 [22.4%] of 510), HNSC (205 [39.7%] of 517) and OV (132 [23.5%] of 562). Those patients with *PMS2* gene amplification have significantly shorter survival compared to non-CNAmp patients in each cancer type (GBM, HR = 1.53, 95% CI 1.22 to 1.92, *P* = 1.98×10^-4^; LGG, HR = 2.32, 95% CI 1.69 to 3.19, *P* = 2.33×10^-7^; HNSC, HR = 1.58, 95% CI 1.24 to 2.02, *P* = 2.22×10^-4^; OV, HR = 1.42, 95% CI 1.12 to 1.79, *P* = 3.48×10^-3^) (**Figure 3B**). Another recurrently amplified DDR gene, *POLM*, plays an essential role in NHEJ repair for DSB 34, 35. Poor survival was observed in patients with *POLM* amplification in multiple cancer types (HNSC: CNAmp frequency = 36.4% [188 of 517], HR = 1.40, 95% CI 1.09 to 1.81, *P* = 9.17×10^-3^; LGG: CNAmp frequency = 23.5% [120 of 510], HR = 2.47, 95% CI 1.78 to 3.44, *P* = 6.83×10^-3^; and LUAD: CNAmp frequency = 51.9% [265 of 511], HR = 1.52, 95% CI 1.15 to 2.03, *P* = 3.83×10^-3^) (**Figure 3C**). *PRKDC* amplification also significantly correlated with poor patient survival in multiple cancer types (sarcoma [SARC]: CNAmp frequency = 35.2% [89 of 253], HR = 2.15, 95% CI 1.46 to 3.18, *P* = 1.12×10^-4^; uterine corpus endometrial carcinoma [UCEC]: CNAmp frequency = 31.5% [165 of 523], HR = 1.72, 95% CI 1.21 to 2.44, *P* = 2.50×10^-3^) (**Figure 3D**). Results for more DDR genes can be found in **Figure S3B**.

### Integrated evidence revealed that *NBN* CNAmp induces cisplatin and PARP inhibitor resistance in breast and ovarian cancer

**Figure 4:**
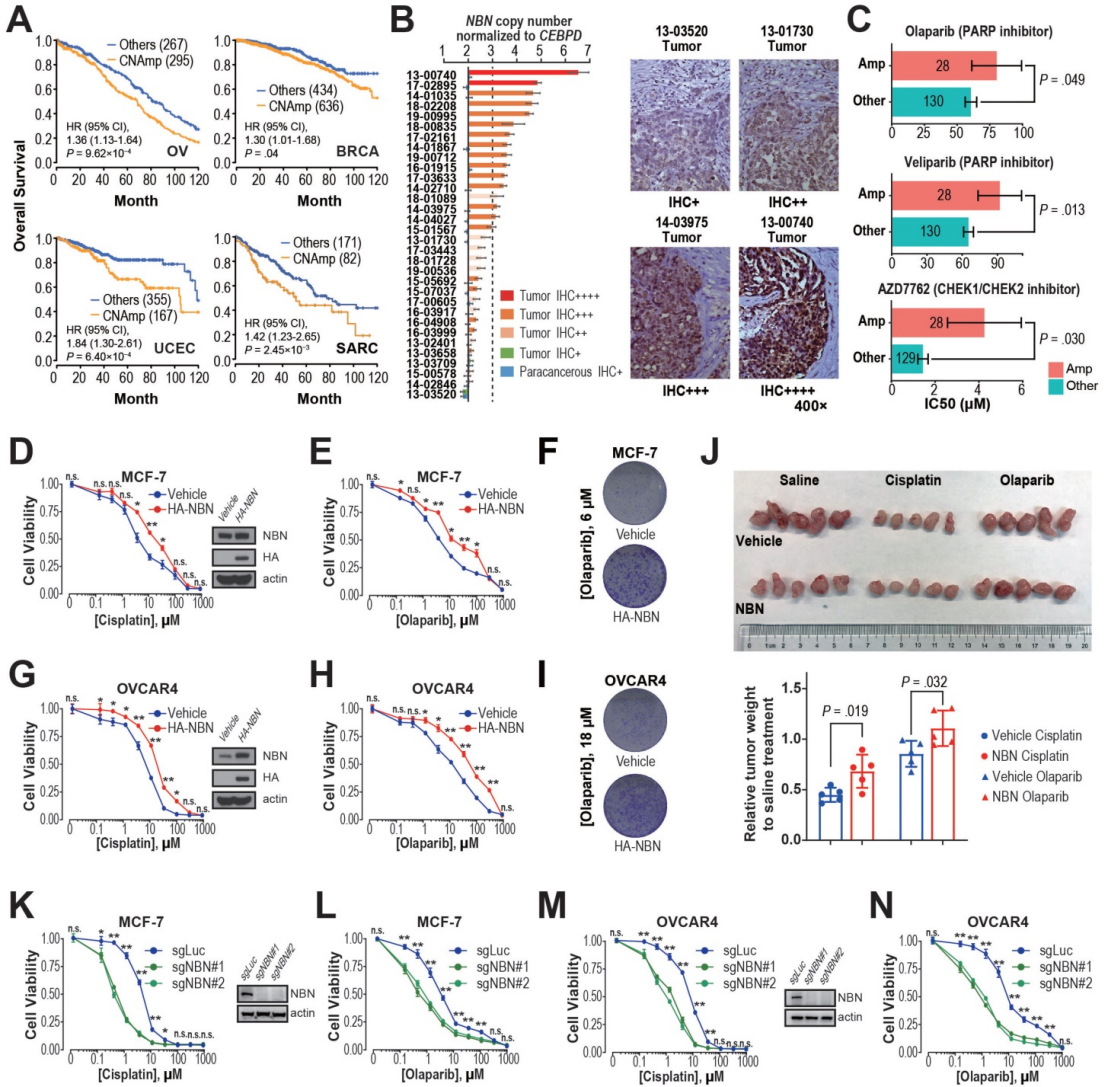
***NBN* amplification induces cisplatin and PARPi resistance in breast and ovarian cancer. A**) Kaplan-Meier curves for OV, BRCA, UCEC, and SARC patients with or without *NBN* CNAmp. Overall survival rates were compared by Cox regression model using the DDR gene copy number SCNA score. **B**) NBN protein is overexpressed (determined by immunohistochemistry) in the *NBN* amplified (determined by digital droplet PCR) patient samples. Malignant/para-cancerous tissues obtained from 32 serous epithelial ovarian cancer cases (Fisher's exact test, *P* = 0.012). **C**) Cancer cell lines with *NBN* amplification (GDSC copy number score ≥ 5) show increased drug IC50 to PARP inhibitors (olaparib and veliparib) and CHECK1/2 inhibitor (AZD7762) in the GDSC database. Error bars indicate mean ± SEM. **D-F**) *NBN* overexpression promotes resistance to cisplatin (**D.** MTT assay) and olaparib (E**.** MTT assay; **F.** clonogenic assay) treatment in MCF-7 cells. **G-I**) *NBN* overexpression promotes resistance to cisplatin (**G.** MTT assay) and olaparib (**H.** MTT assay; **I.** clonogenic assay) treatment in OVCAR4 cells. **J**) Representative tumor size (upper), and relative quantification of tumor weight (lower) from xenograft mouse drug response models. Data are presented as mean ± SD (n = 5). **K, L**) *NBN* knockout by lentiCRISPR/Cas9 sensitizes MCF-7 cells to cisplatin (**K**) and olaparib (**L**) treatment. **M, N**) *NBN* knockout by lentiCRISPR/Cas9 system sensitizes OVCAR4 cells to cisplatin (**M**) and olaparib (**N**) treatment. For the MTT assay, the cell viability data are presented as mean ± SEM (n = 3 for technical replicates). n.s.: not significant, *: *P* < 0.05, **: *P* < 0.005.

Previous studies have demonstrated that restored DDR function leads to chemotherapy resistance and thus poor patient survival 15, 36, 37. The observation of significant positive correlations between DDR gene CNAmp and reduced tumor mutation burden, mechanism specific mutation signatures, and poor patient survival lead to our hypothesis that CNAmp of these DDR genes may cause poor patient survival by augmenting DDR function and consequently chemotherapy resistance in the tumor. Among the recurrent DDR gene CNAmps, *NBN* CNAmp is the most prominent molecular event that occurs in over 40% of patients across 16 cancer types. *NBN*'s overexpression is highly driven by its CNAmp in both primary tumors (TCGA database, *P* = 2.50×10^-60^) and cancer cell lines (GDSC database, *P* = 3.94×10^-5^), which was also observed in two independent serous ovarian carcinoma studies (Etemadmoghadam D, et al.^38^, ^39^, *P* = 2.45×10^-7^; Ducie J, et al.^40^, *P* < 10^-5^) (**Figure S3C**). Moreover, *NBN* CNAmp is most prominently correlated with poor overall survival in OV patients (HR = 1.36, 95% CI 1.13 to 1.64, *P* = 9.62×10^-4^) (**Figure 4A**). To corroborate *NBN*'s CNAmp and overexpression in ovarian cancer, we further experimentally quantified *NBN* gene copy number and protein expression in an independent cohort of 31 serous ovarian cancer samples using droplet digital PCR and immunohistochemistry respectively. These assays independently validated that *NBN* protein overexpression is highly associated with its CNAmp (Fisher's exact test, *P* = 0.012) (**Figure 4B**).

**Figure 5:**
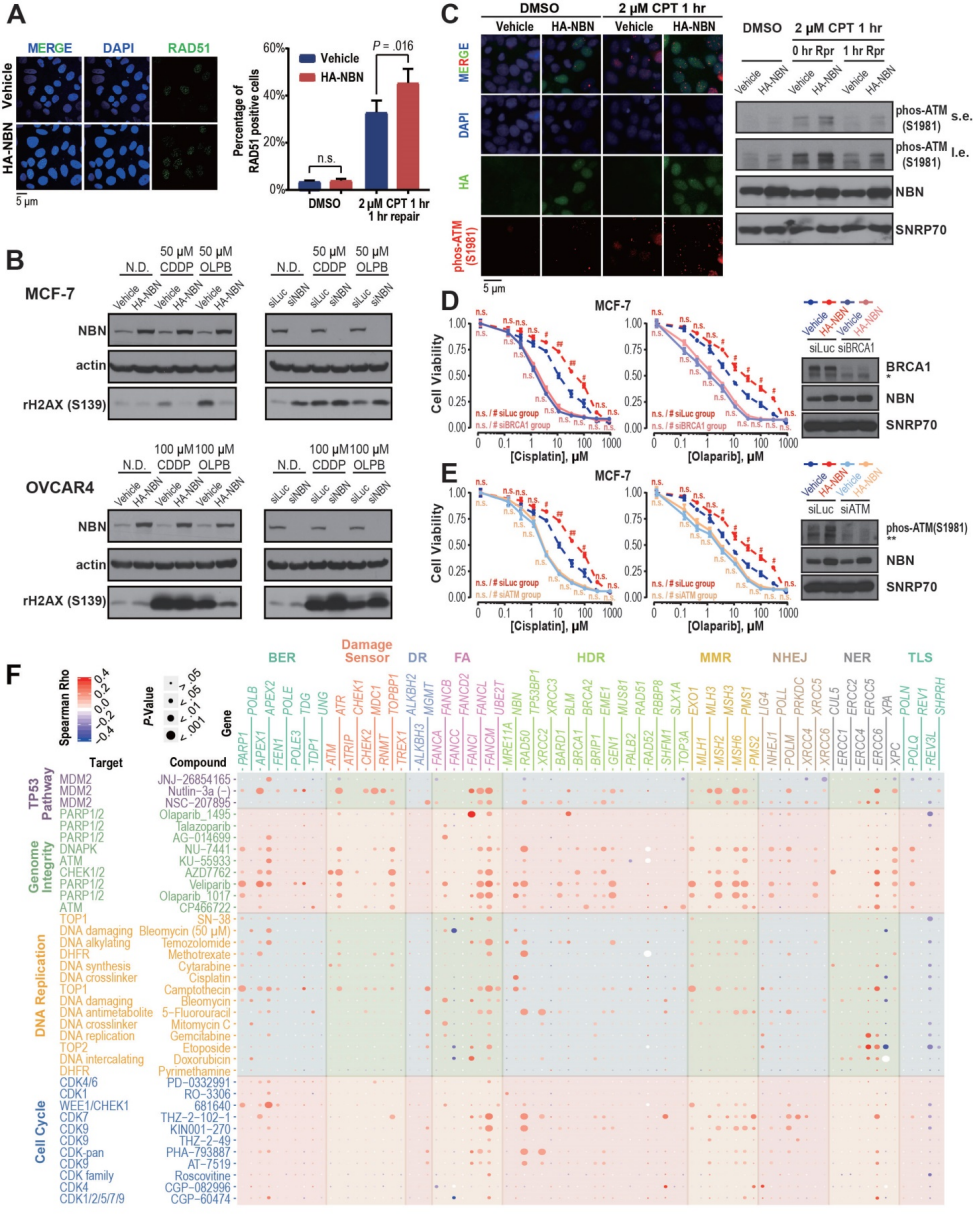
** DDR gene amplification is associated with cell line drug resistance. A**) *NBN* overexpression significantly (Student's* t*-test, *P* = 0.016) increases RAD51 foci formation after camptothecin (CPT) treatment in MCF-7 cells. **B**) *NBN* overexpression (or knockdown) decreases (or increases) the γH2AX expression after double-strand break drug treatment in MCF-7 and OVCAR4 cells. N.D.: no drug control; CDDP: cisplatin; OLPB: olaparib. **C**) *NBN* overexpression increases p-ATM (Ser1981) foci formation and phosphorylated protein expression after camptothecin treatment in MCF-7 cells. Rpr: drug-free repair in fresh medium. s.e.: short exposure. l.e.: long exposure. **D**) *BRCA1* or *ATM* (**E**) knockdown rescues the *NBN* overexpression-induced cisplatin and olaparib resistance in MCF-7 cells (MTT assay). * non-specific band of BRCA1 antibody. ** non-specific band of p-ATM antibody. Cell viability data are presented as mean ± SEM (n = 3 for technical replicates). n.s.: not significant, ^#^: *P* < 0.05, ^##^: *P* < 0.005. **F**) DDR gene copy number is highly associated with the *in vitro* response of drugs targeting genome-instability. The Spearman's rank correlations test was used to determine the correlation between the copy number alteration of each of the 80 core DDR genes and the log-transformed IC50 of the 37 genome-instability targeting drugs across the cancer cell lines. The color and size of each bubble indicate the Spearman's rank correlation coefficients and *P*-values, respectively.

The *NBN* gene encodes Nibrin (P95), a component of the MRE11-RAD50-NBN (MRN) complex that has been identified as a crucial player in the DSB end processing and HDR repairing processes 41. Since platinum-based drugs and PARP inhibitors have been extensively used as the DSB-targeting chemotherapy of ovarian cancer, we performed a correlation analysis between *NBN* copy number alterations and the drug treatment response to cisplatin and PARPis across 505 cancer cell lines from the GDSC database. This analysis revealed that *NBN* copy number is highly correlated with cellular viability responses to cisplatin treatment (*rho* = 0.25, *P* = 1.60×10^-3^), which is the most commonly used chemotherapy for OV patients. Moreover, the cancer cell lines with *NBN* amplification showed significantly increased resistance to PARP inhibitors olaparib and veliparib (**Figure 4C**). We thus overexpressed *NBN* in OVCAR4 and MCF-7 cells. MTT assay and clonogenic assay indicated that *NBN* over-expression can significantly increase resistance to cisplatin and olaparib in MCF-7 (**Figure 4D**, **E**, **and F**) and OVCAR4 (**Figure 4G**, **H**, **and I**) cells. The increased resistance to cisplatin and olaparib after *NBN* overexpression was also confirmed *in vivo* in a xenograft drug response mouse model (cisplatin, *P* = 0.019; olaparib, *P* = 0.032) (**Figure 4J**). Consistently, *NBN* depletion significantly sensitized these cancer cells to cisplatin or olaparib treatment (**Figure 4K-N** and **Figure S3D**). In accordance with its regulation of cell line response to cisplatin and olaparib treatments, *NBN* overexpression significantly augmented HDR proficiency as indicated by increased RAD51 foci formation after DSB induction by camptothecin (**Figure 5A**) and reduced γH2AX phosphorylation after cisplatin or olaparib treatment (**Figure 5B**) in both MCF-7 and OVCAR4 cells.

It has been well documented that the MRN complex interacts with ATM during the initial stage of HDR 42. Our analysis also found a strong association between *NBN* amplification and AZD7762 (inhibitor for ATM substrate CHECK1/2) resistance (**Figure 4C**). After DSB induction by camptothecin, MCF-7 cells overexpressing *NBN* showed an increased level of ATM phosphorylation (S1981) compared with the control cell line (**Figure 5C**), suggesting that *NBN* CNAmp's induction of chemotherapy resistance may be mediated by ATM phosphorylation. ATM phosphorylation is the critical step for the activation of downstream HDR proteins including BRCA1 43, 44. Further siRNA treatment of either the *ATM* or the *BRCA1* gene alleviates the *NBN* overexpression induced drug (e.g., cisplatin and olaparib) resistance phenotype in MCF-7 cells (**Figure 5D**, **E**), which unequivocally demonstrates that the *NBN* CNAmp induced drug-resistant phenotype is mediated through the ATM-HDR activation.

### Pharmacogenomics analysis unveiled an overall significant correlation between DDR gene CNAmps and genome-instability targeting drug resistance

Resistance to chemotherapy is one of the major barriers to improving cancer survival. One-third of the current FDA approved anti-cancer drugs are targeting genome instability or DNA replication 45. Encouraged by the experimental validation that *NBN* CNAmp leads to cisplatin and olaparib resistance, we wondered if other DDR gene CNAmps could lead to poor patient prognosis through prompting resistance to genome instability targeting chemotherapy. In this regard, we further integrated the copy number alterations data across 505 cancer cell lines and their responses to 37 genome-instability targeting drugs (i.e., 23 DNA-damaging drugs and 14 cell cycle/TP53 targeting agents) in GDSC. This analysis revealed the landscape of DDR gene CNAmps and response to genome-instability targeting drugs including 468 significant associations between the 80 DDR CNAmps and the 37 genome-instability targeting drugs (**Figure 5F**). Among the 468 significant associations, 430 (92%) significant positive correlations indicated that DDR gene CNAmp lead to drug resistance (**Table S4**), suggesting that the CNAmp of DDR genes might induce a resistant phenotype to chemotherapy targeting genome-instability.

Gene-level analysis revealed that CNAmp of *FANCM*, the pivotal component of the Fanconi anemia (FA) pathway that relieves the DNA inter-strand cross-link 46, has significant correlations with cellular responses to DSB inducing agents such as topoisomerase inhibitor (camptothecin, *P* = 5.54×10^-3^), CHECK1/2 inhibitor (AZD7762, *P* = 1.90×10^-5^), and PARP inhibitors (olaparib: *P* = 3.34×10^-3^, veliparib: *P* = 1.29×10^-4^) (**Table S4**), which is consistent with previous studies showing that *FANCM* is intensively involved in the DDR response and regulates PARPi sensitivity 47,48.

On the pathway level, the cancer cell lines with CNAmp of DDR genes across the BER (5 out of 10 genes), FA (3 out of 8 genes) and HDR (7 out of 21 genes) pathways exhibited significantly higher IC50s to camptothecin (*P* < 0.05). Camptothecin (Irinotecan) is a topoisomerase inhibitor that induces lethal replication fork collisions between advancing replication forks during S-phase DNA replication. The BER, FA and HDR pathways are critical for the rescue of the stalled replication fork 49-52, which provides a mechanistic explanation for the correlation between DDR pathway amplification and Irinotecan resistance.

Consistent with our previous observation, HDR pathway CNAmp is associated with increased IC50 of PARPi. Six (29% of 21) HDR genes (*NBN*, *GEN1*, *BARD1*, *RAD50*, *BRCA1*, and *BRIP1*) showed significant positive correlations between the gene copy number alterations and cell line responses to veliparib and olaparib treatments respectively (*P* < 0.05) (**Table S4**). Together with our previous experimental phenotype validation of *NBN* overexpression in the cancer cell lines, this study further suggests that the observed significant correlation between DDR gene CNAmp and poor patient survival maybe attributed to increased DDR function and chemotherapy resistance in tumors with DDR gene CNAmps.

## Discussion

In the current study, by integrating multi-dimensional genomics and clinical data in cancer patients and cancer cell lines, we demonstrated that DNA damage repair (DDR) genes' copy number amplification/gain (CNAmp) and overexpression not only recurrently occur across 32 cancer types, but also lead to elevated DNA repair capacity and increased chemotherapy resistance. To the best of our knowledge, this is the first study systematically depicting the DDR pathways copy number amplification landscape and their clinical consequences in cancer.

DDR pathway deficiencies have been well-established as drug-actionable targets for cancer therapy 53,54. However, frequent CNAmp of DDR genes, which may play critical roles in the therapeutic resistance of cancer, have long been neglected in cancer study and treatment. Previous studies reported that the restoration of homology-dependent recombination (HDR) function by somatic reversion of germline *BRCA1/2* mutations confers platinum and PARPi resistance in ovarian cancer 37. In our study, we have unveiled a general connection between DDR gene CNAmps, increased genome integrity, and poor cancer patient survival. Our analysis revealed that the DDR genes are significantly overexpressed in tumors with DDR CNAmps, and correlates with the reduced genome instability across 32 cancer types. Since the genome instability has been intensively reported as a prognosis marker for the cancer patient 55-57, we further adjusted the survival analysis using the genome instability data and confirmed that the strong connection between DDR gene amplification and poor patient survival still stood (data not shown).

Consistent with their association with poor prognosis, DDR gene amplification were shown to be correlated with anti-cancer drug resistance to 37 genome instability-targeting drugs across 505 cancer cell lines. We have further experimentally validated that overexpression of *NBN*, the most frequently amplified DDR gene in the pan-cancer cohort; can directly induce the olaparib resistance in both breast and ovarian cancer cell lines through activating the HDR pathway. Whether the *NBN* overexpression induced cisplatin and olaparib resistance is specifically mediated by BRCA1 will be studied in the future investigation. Our study has not only provided a panorama of DDR genes and anti-cancer drugs interactions, but also a novel molecular mechanism for the intrinsic resistance of genome-instability targeting chemotherapy. Note that all the 10,489 tumor samples and 505 cancer cell lines in this study are chemotherapy naïve, which means that the DDR gene CNAmps have already existed in some tumors before chemotherapy. It is possible that some patients have a subclone of cancer cells with DDR gene CNAmp in their primary tumors. Those patients will initially respond to DDR targeting drugs, and later on develop resistance to the drug after a DDR gene CNAmp subclone starts to expand and becomes the major clone under drug selection pressure 58. One limitation of our study was that we were unable to determine if DDR gene CNAmp occurs in all cancer cells or only a subclone within a tumor. Further study harnessing single-cell sequencing technology 59, 60, and clinical follow-ups are required to comprehensively decipher the roles of DDR gene CNAmp events in cancer development and drug resistance.

DDR gene CNAmps may also serve as reliable biomarkers for cancer precision therapy. Tremendous efforts have been invested to develop clinical actionable methods to measure DDR function and thus predict clinical outcome and chemotherapy response in various cancer types. Those methods include HDR assay, DDR gene-expression profiling, and HDR protein quantification 61-63. However, poor technical reproducibility and inconsistent results with current DDR signaling models raise the translational barrier for these conceivable markers 64, 65. In this study, we have confirmed the *NBN* CNAmp by digital droplet PCR and its strong correlation with gene overexpression in serous ovarian cancer samples. These findings suggest that the copy number quantification of DDR genes at the DNA level can be a promising biomarker for beneficiary identification, response evaluation and prediction of genome instability-targeting therapy, although the clinical feasibility awaits validation from further clinical trials.

## Supplementary Material

Supplementary figures and tables.

## Abbreviations

DDR: DNA damage repair
PARPi: poly ADP ribose polymerase inhibitor
HDR: homology-dependent recombination
GDSC: genomics of drug sensitivity in cancer
GSEA: gene set enrichment analysis
TCGA: the cancer genome atlas
CNAmp: copy number amplification/gain
FFPE: formalin fixed paraffin embedded
ddPCR: digital droplet PCR
BER: base excision repair
DR: direct repair
FA: Fanconi anemia
MMR: mismatch repair
NHEJ: non-homologous end joining
NER: nucleotide excision repair
TLS: translesion synthesis
DSB: double strand break
LUAD: lung adenocarcinoma
READ: rectum adenocarcinoma
OV: ovarian serous cystadenocarcinoma
LGG: lower grade glioma
GBM: glioblastoma multiforme
BRCA: breast invasive carcinoma
COAD: colon adenocarcinoma
HNSC: head and neck squamous carcinoma
ESCA: esophageal carcinoma
LIHC: liver hepatocellular carcinoma
CHOL: cholangiocarcinoma
SKCM: skin cutaneous melanoma
SARC: sarcoma
