# Supplementary material for: Copy Number Amplification of DNA Damage Repair Pathways Potentiates Therapeutic Resistance in Cancer
Source: Theranostics. 2020 Mar 4;10(9):3939–51. doi: 10.7150/thno.39341 (PMC7086350; doi:10.7150/thno.39341)
Supplement: Supplementary file 1 — Supplementary figures and tables. [file thnov10p3939s1.pdf]

Supplementary Online Content

Copy Number Amplification of DNA Damage Repair Pathways Potentiates Therapeutic Resistance in Cancer

Supplementary Methods

References

Figure S1 Overview of DDR core genes amplification across pan-cancer

Figure S2 DDR gene amplified tumors harbor reduced genome instability

Figure S3 DDR gene copy number CNAmp in the tumor correlates with poor patient survival

Table S1 Summary of the copy number alteration of 80 core-list DDR genes in TCGA PanCanAtlas cohort (n = 10,489)

Table S2 Recurrently amplified or deleted DDR genes in TCGA PanCanAtlas cohort (n = 10,489)

Table S3 DDR gene copy number alteration correlates with mRNA expression across TCGA PanCanAtlas patient samples and GDSC cancer cell lines

Table S4 DDR gene copy number alteration correlates with the drug response (IC50) of 37 genome-instability targeting drugs in 505 GDSC cancer cell lines

Supplementary Methods

Genomic and Transcriptomic Data from PanCanAtlas

RNA-Seq gene expression and somatic copy number alteration (SCNA) of 80 "core-list" from 276 "full-list" DNA Damage Repair (DDR) genes [1, 2] in 10,489 cancer patients of 32 cancer types were collected from the National Cancer Institute (NCI) Genomic Data Commons (GDC) legacy archive [3] and the PanCanAtlas publication page [4]. The copy number segmentation data were obtained from the Circular Binary Segmentation (CBS) [5] algorithm, and used for the correlation analysis. The copy number alteration calls, including -2 (deletion), -1 (loss), 0 (diploid), 1 (gain), and 2 (amplification) (Supplement 2), were made from GISTIC2.0 [6] method on the pan-cancer patient samples. The tumor mutation information, including mutation burden, mutation signatures for the patient samples were obtained from the cancer genome atlas (TCGA) database [7].

Patient Clinical Chemotherapy, Radiotherapy and Survival Information

The chemotherapy, radiotherapy and patient survival information [8] of 10,237 TCGA patients across 33 cancer types were obtained from the GDC using the R package TCGAbiolinks [9] with further cleaning and correction.

Cancer Cell Line Gene Copy Number Alteration, Expression and Drug Response Data

For cancer cell lines, the copy number alteration and mRNA expressions of the 80 core DDR genes were downloaded from the Genomics of Drug Sensitivity in Cancer (GDSC) [10]. Drug response data of 265 agents including 37 genome-stability targeting drugs across 1 005 cancer cell lines were downloaded from the GDSC database and processed as in our previous report [11], with 505 cell lines with multi-dimensional genomic and drug treatment response data available retained for the following analysis. The logarithmic transformed half maximal inhibitory concentration (IC50) value was used to indicate the drug response in each cell line.

Summary for DDR Pathway Alteration across PanCanAtlas

Recurrently amplified/deleted DDR genes: Genes with over 5% of samples harboring GISTIC score = -2 or 2 in more than two cancer types were defined as recurrently copy number deleted or amplified.
Pathway level DDR amplification: A pathway is called amplified in one sample if at least one gene in the pathway showed amplification in the sample.

Association Analysis between DNA Repair Gene Copy Number Alteration and mRNA Expression

Spearman's rank correlation coefficient was used to detect the correlation between the gene expression and copy number alteration for each gene in the cell lines and patient samples respectively. Gene Set Enrichment Analysis (GSEA) [12] was performed based on the protein-coding gene list ranked by the signed log transformed Spearman's rank correlation *P*-values using different DDR pathway gene sets from the DDR "full-list" to further interpret the association between the DDR gene amplification and mRNA overexpression.
To further confirm the correlation between *NBN* expression and copy number variations serous ovarian carcinoma, we obtained *NBN* gene expression and copy number variation data of two independent studies (GSE13813/GSE9891 [13, 14] and GSE102094 [15]) from Gene Expression Omnibus (GEO), and performed Spearman's rank correlation analysis for the gene expression and copy number alteration.

Association Analysis between DDR Gene Copy Number Alteration and Genome Mutation Burden and Mutational Signatures

Two-sample *t*-test was used to assess the difference in mutation burden/mutation signature scores between samples containing copy number amplifications (GISTIC score = 2) of a specific gene vs. the other samples. So as to identify the mutation burden reduction related DDR pathway amplification event, Wilcoxon rank-sum test was done for each gene to compare the mutation burden between amplified samples (GISTIC score = 2) and other samples in a cancer-specific manner. Then GSEA was performed based on the protein-coding gene list ranked by the signed log transformed Wilcoxon rank-sum test *P*-values using the full-list DDR pathway genes.

Survival Analysis

The overall survival rates were estimated by Kaplan-Meier analysis to assess differences in survival between patient groups stratified by the DDR gene GISTIC calls in each specific cancer type. Cox proportional hazards regression model was used to detect the correlation of DDR gene copy number alterations with overall patient survival rates.

Association Analysis between DDR Gene Copy Number Alteration and Cell Line Drug Response

The correlation between the copy number alteration of individual genes and treatment responses for each drug was determined by Spearman's rank correlation coefficient. The difference between drug responses in the cell lines bearing different DDR gene copy number alteration was identified by Wilcoxon rank-sum test.

Antibodies

Antibodies used for immunofluorescence and immunoblotting were purchased from the manufacturers as follows. Primary antibodies: phosphorylated ATM (S1981) (Cell Signaling Technology, #4526), BRCA1 (Santa Cruz Biotechnology, #sc-6954), NBN (Abcam, #ab32074), HA (Santa Cruz Biotechnology, #sc-805), γH2AX (Abcam, #ab266350), RAD51 (Protein Atlas, #HPA039310), beta-actin (Sigma-Aldrich, #A5316), SNRP70 (Abcam, #ab51266). Secondary Antibodies: HRP-labeled goat anti-rabbit IgG secondary (Thermo Fisher, #31460), HRP-labeled goat anti-mouse IgG (Thermo Fisher, #31430), Alexa Fluor 488 labeled goat anti-rabbit IgG secondary (Abcam, #ab150077), Alexa Fluor 594 labeled goat anti-mouse IgG secondary (Abcam, #ab150116).

Digital Droplet PCR (ddPCR) for *NBN* Copy Number Quantification in Ovarian Cancer Tissues

FFPE ovarian cancer/para-cancerous tissues were collected from 31 serous epithelial ovarian cancer patients in the Department of Gynecological Surgery in Obstetrics & Gynecology Hospital of Fudan University. Written informed consent was received from all the participants. In compliance with the Helsinki Declaration of 1975 as revised in 1996, this study was approved by both the Institutional Review Boards of the Obstetrics & Gynecology Hospital and Huashan Hospital of Fudan University.

The FFPE tissue samples were digested by incubation overnight in the lysis buffer and proteinase K at 56°C to fully digest the tissues. Genomic DNA (gDNA) was then extracted using the QIAamp DNA FFPE Tissue Kit (Qiagen, #56404) according to the manufacturer's protocol. The gDNA was further fragmented with restriction enzyme (New England BioLabs, #R0136S) digestion for 1 hour.

ddPCR was performed to quantify the gene copy number of *NBN* and chromosome 8 centromere control

CEBPD. For every sample, two ddPCR reactions were scaled in 20 µL volume with 10 µL 2×QX200 ddPCR EvaGreen Supermix (Bio-Rad, #1864033), 10–20 ng of digested gDNA, forward primers (NBN-FP: 5'- GGGAAATATGAATTGTTAGTTG-3' or CEBPD-FP: 5'-TCTACATCTTACTCCTGTTGAT-3') and reverse primers (NBN-RP: 5'-CACTCAGTACATTCACTTC-3' or CEBPD-RP: 5'-CAAATGCTGCTTTATTCTTACAA-3'), each at a final concentration of 500 nM. Droplets were then generated in the QX200 droplet generator (Bio-Rad) by loading 20 µL of the reaction mixture and 70 µL of droplet generation oil for EvaGreen (Bio-Rad, #1864005) onto matched wells of a DG8 cartridge (Bio-Rad). 45 µL of the droplet/oil mixture was peptide to a semi-skirted 96-well plate (Bio-Rad). The plate was sealed with a pierce-able foil heat seal and loaded on a PTC-200 thermal cycler (Bio-Rad). The amplification was performed with the following protocol: 95°C for 10 min, followed by 50 cycles of thermal cycling: denaturation at 94°C for 30 s; annealing at 60°C for 1 min; extension at 65°C for 30 s. The sample was heated at 98°C for 10 min to deactivate the enzyme and then held at 4°C. Cycling between the temperatures was set to a ramp rate of 2.5°C/sec. Upon completion of the PCR protocol, the plate was read using the QX200 droplet reader (Bio-Rad). Droplet counts and amplitudes were then exported to and analyzed with QuantaSoft™ software (Bio-Rad). The copy number of *NBN* was normalized to *CEBPD*.

Immunohistochemistry for NBN semi-quantification in Ovarian Cancer Tissues

FFPE tissue samples were sectioned at 5 µm thickness on a microtome and floated in 40°C distilled water bath, and then transferred onto histological slides (ThermoFisher, #6776214). The slides were further deparaffinized by two times of 5-minute xylene treatment followed by 2 times of 3-minute 100% alcohol treatment and once through 95%, 70%, and 50% alcohols respectively for 3 minutes each. The slides were then treated by 3% H_2O_2 solution in methanol at room temperature for 10 minutes to block endogenous peroxidase activity and then rinsed three times in PBS for 5 minutes. The slides were immersed in 10mM citrate buffer (pH 6.0) in a staining container and incubated at 95°C for 10 min, then cooled down to room temperature for 20 minutes. After three times of 5-mintue rinse in PBS, the slides were incubated with 5% goat serum (ThermoFisher, #PCN5000) in PBS for 1 hour at room temperature, followed by 1:200 diluted NBN primary antibody solution in 5% goat serum overnight at 4°C. The slides were rinsed three times for 5 minutes and then incubated with Ready to use Biotinylated Goat anti-Rabbit IgG (Abcam, #ab64256) solution for 15 minutes at room temperature, followed by PBS rinse for five times. Streptavidin peroxidase complex (Abcam, #ab64269) was applied to the section and incubated for 10 minutes at room temperature and rinsed by PBS for five times. The visualize reaction was carried out in Diaminobenzidene chromogen (Abcam, #ab64238) solution room temperature incubation for 1 minute and PBS rinse for 5 times. The slides were counterstained with Harris hematoxylin (ThermoFisher, #6765001), followed by serial dehydration and xylene treatment, and sealed with resinence. The stained histological sections were independently reviewed by two pathologists and rated for the grade of NBN staining ranging from + to ++++.

Fisher's exact test was performed to test whether the tumors bearing *NBN* amplification (normalized *NBN* copy ≥ 3) have significantly increased NBN protein expression compared to those *NBN* non-amplified (normalized *NBN* copy < 3) tumors.

Cell Culture and Stable Cell Line Establishment

Human breast cancer cell line MCF-7 was a kind gift from Dr. Shilpa Sant and cultured in Dulbecco's Modified Eagle's medium with 10% fetal bovine serum (FBS), and 1% penicillin, 1% streptomycin (1%

PS). Human ovarian cancer cell line OVCAR4 was purchased from Charles River Laboratories (Fredrick, MD) and cultured in RPMI 1640 medium with 10% FBS and 1% PS. Human ovarian cancer cell line SK-OV3 was purchased from American Type Culture Collection (ATCC) (Manassas, VA) and cultured in McCoy's 5A medium with 10% FBS and 1% PS. Lentiviruses were prepared by HEK (human embryonic kidney) 293T cells (ATCC) transfection with pMD2.G envelope and psPAX2 packaging plasmid together with viral transfer plasmid pLVX-HA-NBN-hygro or pLVX-HA-hygro vehicle for *NBN* overexpression or vehicle control by Lipofectamine™ 2000 (ThermoFisher, #11668019). MCF-7, OVCAR4, and SK-OV3 cell lines were infected by the *NBN* overexpression virus or control vehicle and then selected by hygromycin B (ThermoFisher, # 10687010) treatment (MCF-7, 200 µg/ml; OVCAR4, 60 µg/ml; SK-OV3, 150 µg/ml) for 14 days.

NBN Knockout by lentiCRISPR/Cas9 Gene Targeting

The lentiCRISPR-NT non-targeting control plasmid and lentiCRISPR-NBN#1 (sgRNA: /#2 plasmids targeting *NBN* was prepared with lentiCRISPRv2 (Addgene, #52961). The sequences of the guide RNA target sites are as follows, with the protospacer adjacent motif sequence underlined: 5’-GACGGAGGCTAAGCGTCGCAA-3’ (non-targeting control), 5’-CGAACTTTGAAGTCGGGGGATGG (*NBN* targeting #1), 5’-TTCCCGAACTTTGAAGTCGGGGG-3’ (*NBN* targeting #2). The plasmids targeting *NBN* or non-targeting control were further packed with pMD2.G envelope and psPAX2 packaging plasmid in HEK 293T cells with Lipofectamine™ 2000. MCF-7 and OVCAR4 cell lines were infected by the *NBN* targeting plasmids or control virus followed by puromycin (ThermoFisher, # A1113803) selection (MCF-7, 2µg/ml; OVCAR4, 1 µg/ml) for 7 days.

siRNA Interference

siRNA transfection of MCF-7, OVCAR4 and their derived *NBN* overexpressing and vehicle control cells was undertaken using Lipofectamine RNAiMax (ThermoFisher, #13778150) according to the manufacturer’s instructions. siRNA oligonucleotide master mix targeting *ATM* (sc-29761) and *NBN* (sc-36061) were purchased from Santa Cruz Biotechnology. siRNA targeting *BRCA1* (Slincer™ S457 and S458) was purchased from ThermoFisher (#4390824 and #4390824). siRNA targeting the firefly luciferase gene (sense sequence 5’-CACGUACGCGGAAUACUUCGA-3’) was synthesized by Dharmacon (Lafayette, CO) and used as the negative interference control. All the cancer cell lines subjected to RNA interference were exposed to siRNA for 48 hours before further experimental usage.

Drug Treatment

Olaparib (LC laboratories, #O-9201) and (S)-(+)-camptothecin (Sigma-Aldrich, #C9911) were purchased from the manufacturers and dissolved as DMSO chemical stock. Cisplatin (Selleckchem, #S1166) was purchased and dissolved in PBS stock. Fresh drug solutions were prepared by stock diluting by cell culture medium containing 5% FBS before every treatment, and the untreated control was parallelly prepared to get the final concentration of DMSO or PBS as the drug treating solution.

In vitro Drug Response Assay

The cancer cell lines were passaged by 0.05% trypsin before the day of plating. After overnight recovery, the cells were again trypsinized and seeded to 96 well plates at the final density of 1,500 per well. Untreated medium control and serial dilution of the drug were added to each well after 24 hours. Three well triplications were performed for each drug concentration. The medium and drug solution were

replaced after three days. Five days after first drug administration, the cell viability was determined as cell viability by MTT assay using CellTiter cell proliferation assay kit (Promega, #G4100) according to the manufacturers' instructions.

For the cell line drug response experiment with *BRCA1* or *ATM* RNA interference, the drug treatment duration was reduced to three days since the *BRCA1* or *ATM* knockdown lead to significantly decreased cell proliferation.

Clonal genic assay was performed by seeding cancer cells into 12 well plates at the final density of 10^4 cell per well. The cells were treated with medium control or serial dilutions of the drug in the culture medium for seven days before staining by 0.05% crystal violet in 10% methanol PBS solution.

In-vivo **Xenograft Drug Response Assay**

The 4- to 6-week-old female athymic nude mice (Shanghai Model Organisms Center) were used for the xenograft model. SK-OV3 cancer cell lines with or without *NBN* stable overexpression were trypsinized and washed twice with cold PBS. 0.2 mL PBS containing 5×10^6 cells was subcutaneously injected into the flank of the mice. After two weeks of tumor growth, the mice were further administrated with olaparib (50 mg/kg per day), cisplatin (160 μg/mouse per week) or saline by intraperitoneal injection according to the protocol from Liu, et al. [16]. In keeping with the policy for the humane treatment of tumor-bearing animals, the mice were sacrificed 4 weeks after the drug administration and their tumors were harvested. The drug response was measured as the tumor weight [drug treatment]/tumor weight [saline treatment]. All the animal studies were performed in accordance with the institutional guidelines and project protocols approved by the Institutional Ethics Committee of Huashan Hospital.

Protein isolation and Immunoblotting

Cancer cell samples were harvested with 0.05% trypsin and washed twice with PBS. The pellets were resuspended in RIPA buffer (50 mM Tris-HCl, pH 7.4, 150 mM NaCl, 1 mM EDTA, 0.1% SDS, 1% NP-40, 0.5% sodium deoxycholate, 0.5 mM DTT, 1 mM PMSF, with protease inhibitor cocktail (Sigma, #P8340)) and ice bathed for 30 min.

The cell lysates were diluted with equal volume of 2×SDS sample buffer and resolved by denaturing SDS-PAGE and transferred onto the PVDF membrane (Bio-Rad, #162-0177). The blot was blocked with 5% non-fat milk (LabScientific, #M0841) in PBST at room temperature for 30 minutes and then incubated with primary antibody solution overnight at 4°C. The blot was washed with 10-minute PBST rinsing for three times and then incubated with HRP-labeled secondary antibody solution with agitation for 1 hour at room temperature. After three times of 10-minute rinsing of the blot in PBST, specific bands were visualized by X-ray film exposure with enhanced chemiluminescence (ECL) substrate (ThermoFisher, #32106). The film was finally developed by an AX 700LE film processor (ALPHATEK).

Immunofluorescence and Foci Assay

The genetically modified cancer cells were seeded into the Nunc Lab-Tek II CC^2 chamber slide (ThermoFisher, #154941) at a final density of 2×10^4 per well. After specific culture treatment, the slide was washed with cold PBS and fixed with 4% formaldehyde (ThermoFisher, #F79-1) in PBS. The fixed cell samples were penetrated by 0.25% TritonX-100 (ThermoFisher, #HFH10) for 5 minutes at 4°C and rinsed by PBS for three times. The slide was blocked by 5% goat serum (ThermoFisher, #PCN5000) in PBS and then incubated with 1:100 diluted primary antibody solution in 5% goat serum overnight at 4°C. The slide was then washed for 5 minutes, three times in PBS with agitation and then incubated with the

fluorescent secondary antibody solution at room temperature in the dark. The slide was washed for 5 minutes, three times in PBS and then mounted and sealed with ProLong™ Gold Antifade Mountant with DAPI (ThermoFisher, #P36931).

Immunofluorescence assay was performed on the KEYENCE BZ-X710 fluorescence microscope with BZ-X Viewer software (Keyence) or Olympus FV1000 confocal laser scanning microscope with FV10-AWS software (Olympus). The Foci scoring was carried out blindly with > 800 foci/sample being scored. All foci analysis represents the mean ± SEM of over 10 separated microscope views.

References

1. Wood RD, Mitchell M, Lindahl T. Human DNA Repair Genes. 2014.
2. Knijnenburg TA, Wang L, Zimmermann MT, Chambwe N, Gao GF, Cherniack AD, et al. Genomic and Molecular Landscape of DNA Damage Repair Deficiency across The Cancer Genome Atlas. Cell Rep. 2018; 23: 239-54 e6.
3. The Cancer Genome Atlas. The GDC legacy archive. 2018.
4. The Cancer Genome Atlas. PancanAtlas publication page. 2018.
5. Olshen AB, Venkatraman ES, Lucito R, Wigler M. Circular binary segmentation for the analysis of array-based DNA copy number data. Biostatistics. 2004; 5: 557-72.
6. Mermel CH, Schumacher SE, Hill B, Meyerson ML, Beroukhim R, Getz G. GISTIC2.0 facilitates sensitive and confident localization of the targets of focal somatic copy-number alteration in human cancers. Genome Biol. 2011; 12: R41.
7. Ellrott K, Bailey MH, Saksena G, Covington KR, Kandoth C, Stewart C, et al. Scalable Open Science Approach for Mutation Calling of Tumor Exomes Using Multiple Genomic Pipelines. Cell Syst. 2018; 6: 271-81 e7.
8. Liu J, Lichtenberg T, Hoadley KA, Poisson LM, Lazar AJ, Cherniack AD, et al. An Integrated TCGA Pan-Cancer Clinical Data Resource to Drive High-Quality Survival Outcome Analytics. Cell. 2018; 173: 400-16 e11.
9. Colaprico A, Silva TC, Olsen C, Garofano L, Cava C, Garolini D, et al. TCGAbiolinks: an R/Bioconductor package for integrative analysis of TCGA data. Nucleic Acids Res. 2016; 44: e71.
10. Iorio F, Knijnenburg TA, Vis DJ, Bignell GR, Menden MP, Schubert M, et al. A Landscape of Pharmacogenomic Interactions in Cancer. Cell. 2016; 166: 740-54.
11. Wang Y, Wang Z, Xu J, Li J, Li S, Zhang M, et al. Systematic identification of non-coding pharmacogenomic landscape in cancer. Nat Commun. 2018; 9: 3192.
12. Subramanian A, Tamayo P, Mootha VK, Mukherjee S, Ebert BL, Gillette MA, et al. Gene set enrichment analysis: a knowledge-based approach for interpreting genome-wide expression profiles. Proc Natl Acad Sci U S A. 2005; 102: 15545-50.
13. Etemadmoghadam D, deFazio A, Beroukhim R, Mermel C, George J, Getz G, et al. Integrated genome-wide DNA copy number and expression analysis identifies distinct mechanisms of primary chemoresistance in ovarian carcinomas. Clin Cancer Res. 2009; 15: 1417-27.
14. Tothill RW, Tinker AV, George J, Brown R, Fox SB, Lade S, et al. Novel molecular subtypes of serous and endometrioid ovarian cancer linked to clinical outcome. Clin Cancer Res. 2008; 14: 5198-208.
15. Ducie J, Dao F, Considine M, Olvera N, Shaw PA, Kurman RJ, et al. Molecular analysis of high-grade serous ovarian carcinoma with and without associated serous tubal intra-epithelial carcinoma. Nat Commun. 2017; 8: 990.
16. Liu G, Yang D, Rupaimoole R, Pecot CV, Sun Y, Mangala LS, et al. Augmentation of response to chemotherapy by microRNA-506 through regulation of RAD51 in serous ovarian cancers. J Natl Cancer Inst. 2015; 107.

Figure S1. Overview of DDR core genes amplification across pan-cancer

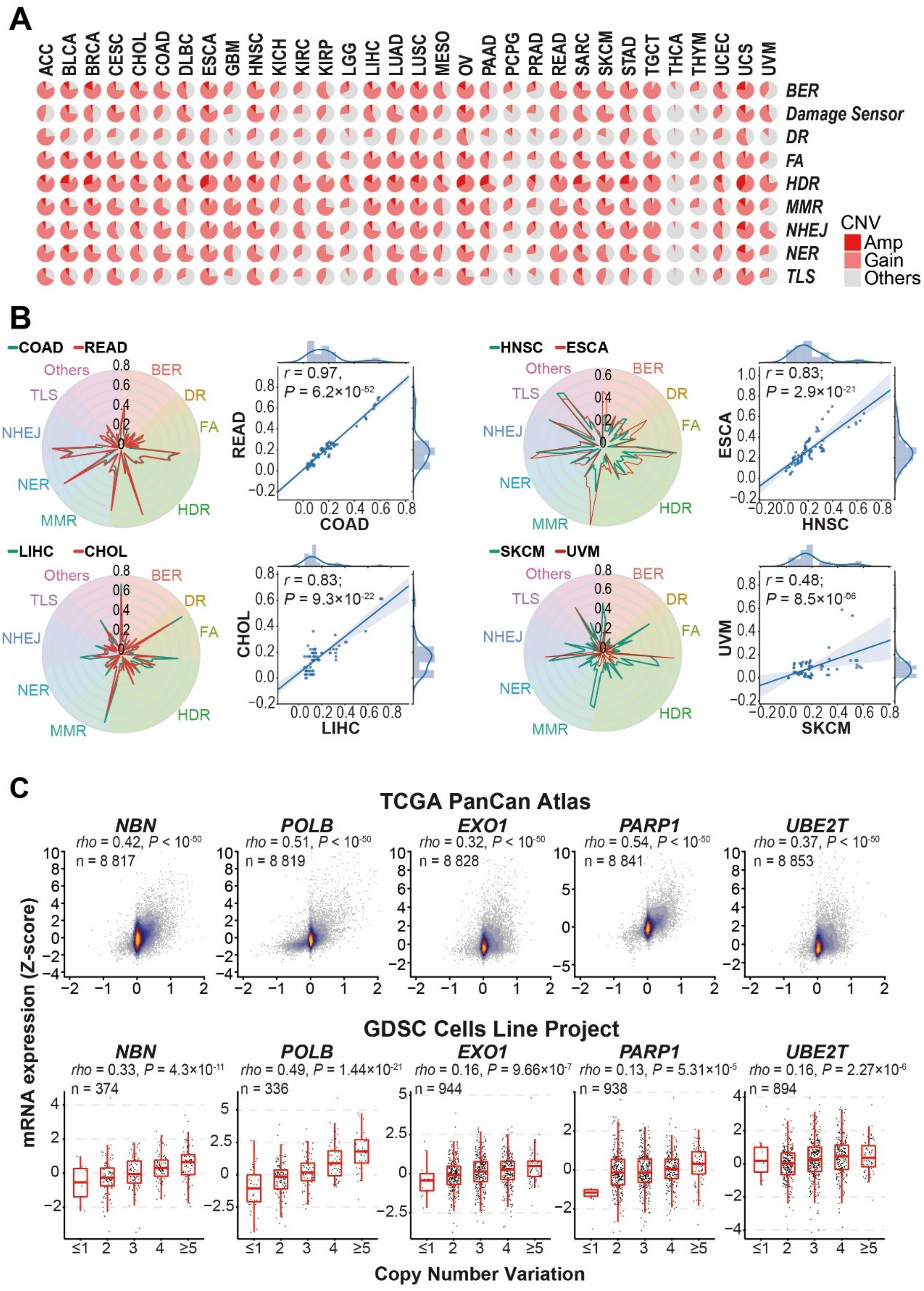

A) Cancer-specific DDR pathway copy number amplification (red) / gain (pink) prevalence. HDR is the most prevalently amplified DDR pathway.

B) Similar gene amplification patterns are found in tumor tissues from the same origin as indicated by the radar plot. The copy number of 80 core DDR genes showed a highly positive correlation in the three tumor pairs with the same

histological origin as COAD-READ, HNSC-ESCA, LIHC-CHOL, but not in SKCM-UVM. Pearson correlation coefficient and *P*-value are indicated.

C) DDR gene expression demonstrated a significant positive correlation with its copy number alteration in the patient samples (top, scatter plot) and cancer cell lines (bottom, jitter plot) respectively. Spearman's rank correlation coefficient and *P*-value are indicated.

Figure S2. DDR gene amplified tumors harbor reduced genome instability

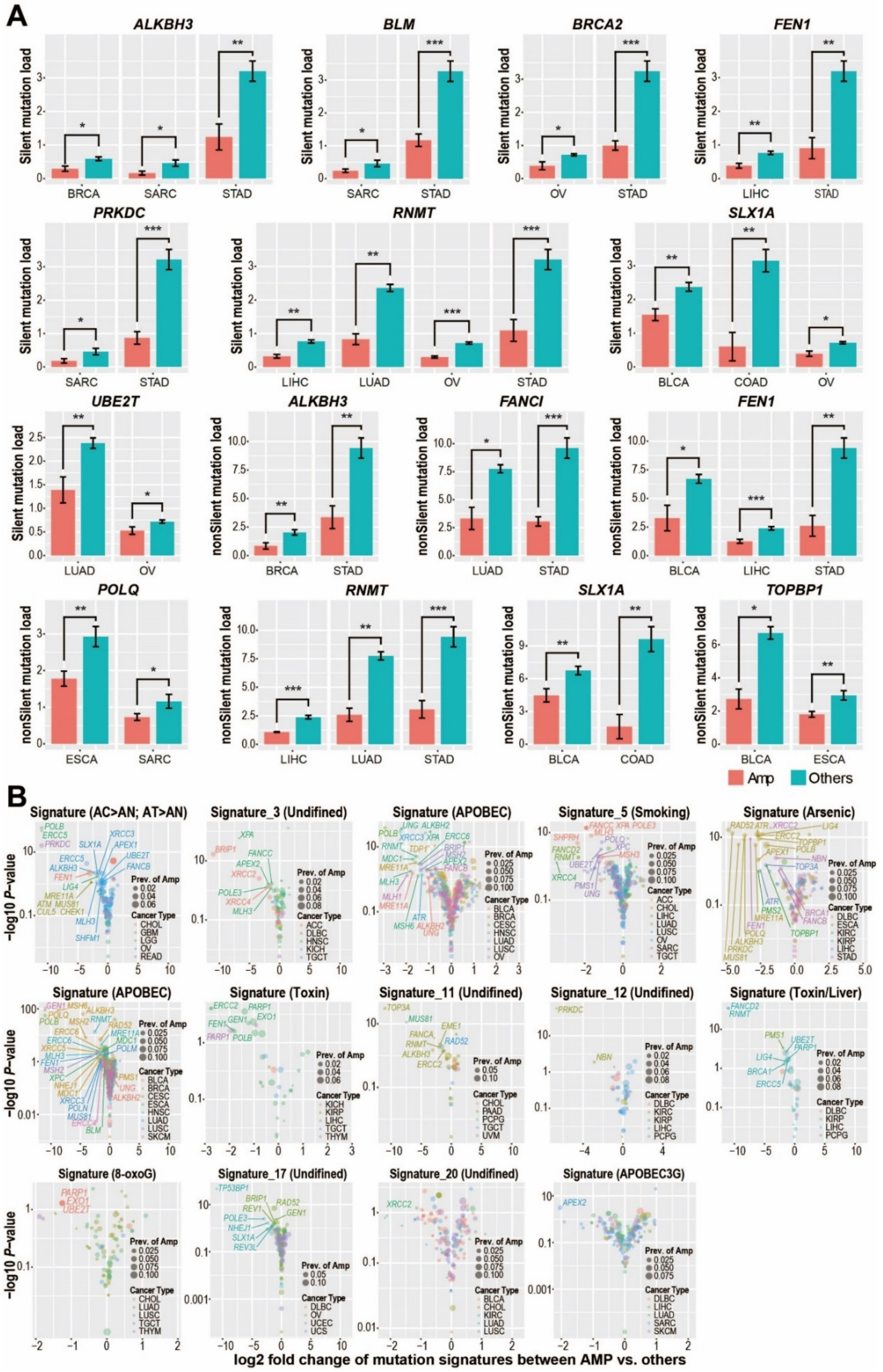

A) Bar chart showing the silent or non-silent mutation burden reduction between tumors with (pink) or without (green) specific DDR gene amplification across multiple cancer types. Median of the mutation burden in each genotype was shown in the bar chart. Error bars indicate mean ± SEM * $P < .05$, ** $P < .005$, *** $P < .0005$. (Student' *t*-test)

B) Volcano plot showing the DDR gene amplification is associated with specific mutational signature reduction.

Figure S3. DDR gene copy number CNAmp in the tumor correlates with poor patient survival and drug resistance

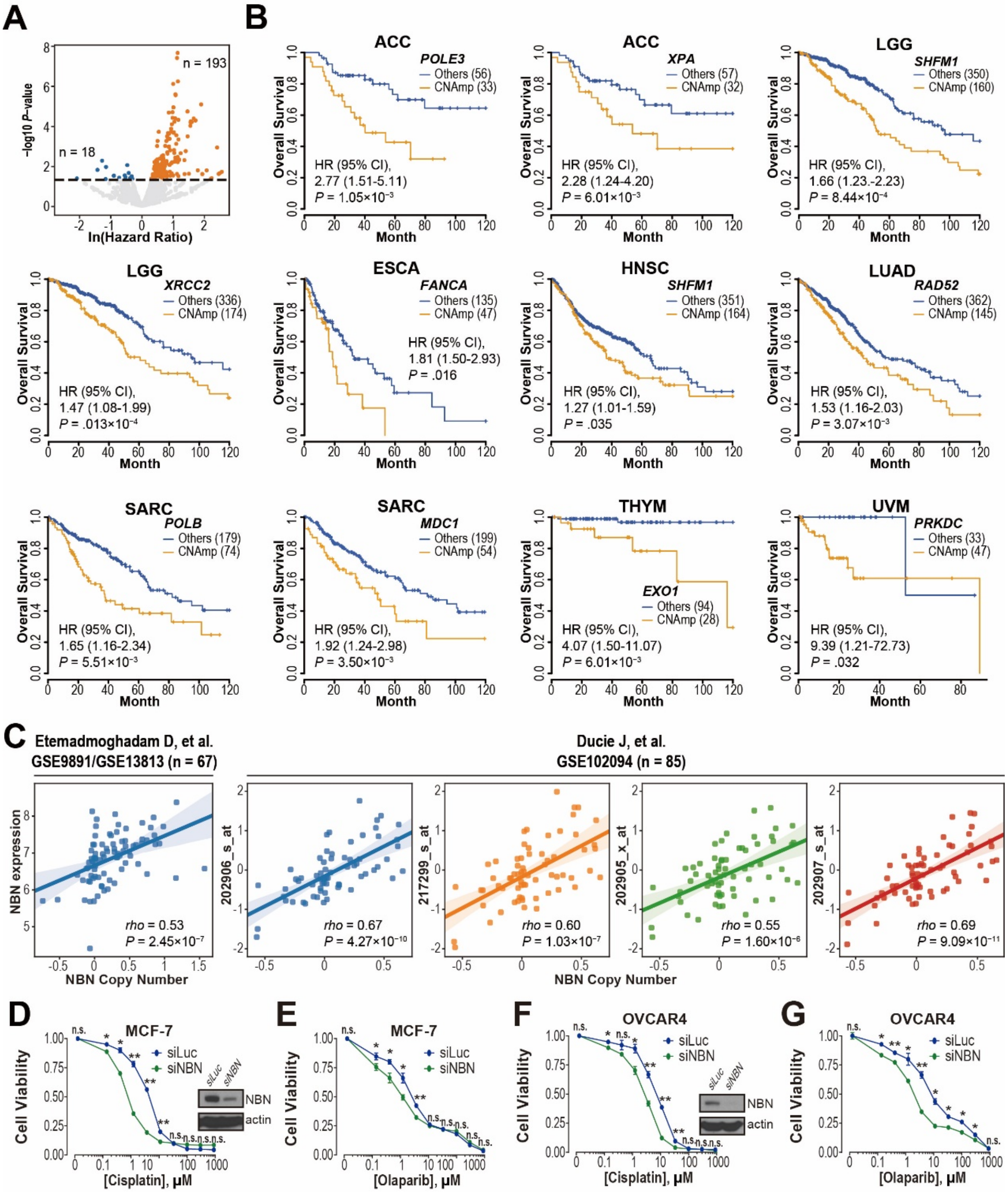

A) Patients bearing tumors with DDR gene amplification showed general poor survival. Volcano plot shows the hazard ratio and *P*-value from Cox regression analysis for the survival of patient with or without specific DDR gene amplification in a specific cancer type. Hazard ratio > 1 means patients mean patients with a specific DDR gene amplification have poor prognosis, compared to patients without the amplification.

B) DDR gene copy number gain/amplification (CNAmp, GISTIC calls = 1 and 2) is associated with patients' poor overall survival (Kaplan-Meier curve with hazard ratio from Cox regression analysis).

C) mRNA expression of *NBN* demonstrated a significant positive correlation with its copy number alteration in the ovarian cancer datasets (GSE13813/GSE9891 and GSE102094). Spearman's rank correlation coefficient and *P*-value are indicated.

D, E) NBN knockdown by siRNA sensitizes MCF-7 cells to cisplatin (D) or olaparib (E) treatment.

F, G) NBN knockdown by siRNA sensitizes OVCAR4 cells to cisplatin (F) or olaparib (G) treatment.

Table S1. Summary of the copy number alterations of 80 core-list DDR genes in TCGA PanCanAtlas cohort (n = 10,489)

Gene Symbol	DDR Pathway	Alias	Gene Description	TCGA_Gain PlusAmp_fre[a]	TCGA_Loss PlusDel_fre[b]
NBN	HDR[f]		Nibrin	0.41	0.06
PMS2	MMR[g]		PMS1 homolog 2, mismatch repair system component	0.38	0.07
POLM	NHEJ[h]		polymerase (DNA) mu	0.38	0.06
SHFM1	HDR[f]	*DSS1, ECD, SEM1, SHFD1, SHSF1*	SEM1, 26S Proteasome Complex Subunit	0.37	0.06
UBE2T	FA[e]	*FANCT*	ubiquitin-conjugating enzyme E2T	0.36	0.06
EXO1	MMR[g]		exonuclease 1	0.35	0.07
PARP1	BER[c]		poly (ADP-ribose) polymerase 1	0.35	0.07
PRKDC	NHEJ[h]		protein kinase, DNA-activated, catalytic polypeptid	0.35	0.09
XRCC2	HDR[f]	*FANCU*	X-ray repair cross complementing 2	0.34	0.11
ATR	Others[k]		ATR serine/threonine kinase	0.28	0.09
TOPBP1	Others[k]		topoisomerase (DNA) II binding protein 1	0.27	0.10
POLB	BER[c]		polymerase (DNA directed), beta	0.27	0.18
RAD52	HDR[f]		RAD52 homolog, DNA repair protein	0.25	0.11
BRIP1	HDR[f]	*BACH1, FANCJ*	BRCA1 interacting protein C-terminal helicase 1	0.25	0.11
POLQ	TLS[j]		polymerase (DNA) theta	0.25	0.11
EME1	HDR[f]		essential meiotic structure-specific endonuclease 1	0.22	0.12
SLX1A	HDR[f]	*GIYD1*	SLX1 homolog A, structure-specific endonuclease subunit	0.21	0.13
ERCC4	NER[i]	*XPF, FANCQ*	excision repair cross-complementation group 4	0.21	0.14
MDC1	Others[k]		mediator of DNA-damage checkpoint 1	0.21	0.13
PALB2	HDR[f]	*FANCN*	partner and localizer of BRCA2	0.20	0.14
MSH2	MMR[g]		mutS homolog 2	0.19	0.08
FANCL	FA[e]		Fanconi anemia, complementation group L	0.19	0.07
MSH6	MMR[g]		mutS homolog 6	0.19	0.08
GEN1	HDR[f]		GEN1 Holliday junction 5' flap endonuclease	0.19	0.09
ERCC1	NER[i]		excision repair cross-complementation group 1	0.18	0.18
ERCC2	NER[i]	*XPD*	excision repair cross-complementation group 2	0.18	0.18
BRCA1	HDR[f]		breast cancer 1, early onset	0.18	0.18
LIG4	NHEJ[h]		DNA ligase 4	0.17	0.26
POLE	BER[c]		polymerase (DNA directed), epsilon, catalytic subunit	0.17	0.15

UNG	BER[c]		uracil-DNA glycosylase	0.17	0.13
ALKBH2	DR[d]		alkB homolog 2, alpha-ketoglutarate dependent dioxygenase	0.17	0.13
TDG	BER[c]		thymine-DNA glycosylase	0.17	0.13
ERCC5	NER[i]	*XPG*	excision repair cross-complementation group 5	0.17	0.26
REV1	TLS[j]	*REV1L*	REV1, DNA directed polymerase	0.16	0.08
PMS1	MMR[g]		PMS1 homolog 1, mismatch repair system component	0.16	0.11
RNMT	Others[k]		RNA guanine-7 methyltransferase	0.15	0.23
MUS81	HDR[f]		MUS81 structure-specific endonuclease subunit	0.15	0.15
APEX1	BER[c]		apurinic/apyrimidinic endodeoxyribonuclease 1	0.15	0.21
BLM	HDR[f]		Bloom syndrome, RecQ helicase-like	0.14	0.20
RBBP8	HDR[f]	*CTIP*	retinoblastoma binding protein 8	0.14	0.23
APEX2	BER[c]		apurinic/apyrimidinic endodeoxyribonuclease 2	0.14	0.18
FANCI	FA[e]	*KIAA1794*	Fanconi anemia, complementation group I	0.14	0.20
XRCC3	HDR[f]		X-ray repair cross complementing 3	0.14	0.24
FEN1	BER[c]		flap structure-specific endonuclease 1	0.13	0.15
RAD50	HDR[f]		RAD50 double strand break repair protein	0.13	0.24
FANCA	FA[e]		Fanconi anemia, complementation group A	0.13	0.30
FANCM	FA[e]		Fanconi anemia, complementation group M	0.13	0.23
BRCA2	HDR[f]		breast cancer 2, early onset	0.13	0.32
FANCB	FA[e]		Fanconi anemia, complementation group B	0.13	0.21
TDP1	BER[c]		tyrosyl-DNA phosphodiesterase 1	0.13	0.25
POLE3	BER[c]		polymerase (DNA directed), epsilon 3, accessory subunit	0.13	0.24
CHEK2	Others[k]		checkpoint kinase 2	0.13	0.28
MRE11A	HDR[f]		MRE11 homolog A, double strand break repair nuclease	0.13	0.22
FANCD2	FA[e]		Fanconi anemia, complementation group D2	0.12	0.28
BARD1	HDR[f]		BRCA1 associated RING domain 1	0.12	0.16
XRCC5	NHEJ[h]	*KU80*	X-ray repair cross complementing 5	0.12	0.16
NHEJ1	NHEJ[h]		nonhomologous end-joining factor 1	0.12	0.16
FANCC	FA[e]		Fanconi anemia, complementation group C	0.12	0.25
MLH3	MMR[g]		mutL homolog 3	0.12	0.26
XPA	NER[i]		xeroderma pigmentosum, complementation group A	0.12	0.25
XPC	NER[i]		xeroderma pigmentosum, complementation group C	0.12	0.28

XRCC6	NHEJ[h]	*KU70*	X-ray repair cross complementing 6	0.12	0.30
ALKBH3	DR[d]		alkB homolog 3, alpha-ketoglutarate dependent dioxygenase	0.11	0.18
CUL5	NER[i]		cullin 5	0.11	0.26
ATM	Others[k]		ATM serine/threonine kinase	0.11	0.26
MSH3	MMR[g]		mutS homolog 3	0.11	0.27
XRCC4	NHEJ[h]		X-ray repair cross complementing 4	0.11	0.27
CHEK1	Others[k]		checkpoint kinase 1	0.11	0.27
MLH1	MMR[g]		mutL homolog 1	0.10	0.29
SHPRH	TLS[j]		SNF2 histone linker PHD RING helicase	0.10	0.27
POLN	TLS[j]		polymerase (DNA) nu	0.10	0.26
TOP3A	HDR[f]		topoisomerase (DNA) III alpha	0.10	0.33
ERCC6	NER[i]	*CSB*	excision repair cross-complementation group 6	0.10	0.25
REV3L	TLS[j]		REV3 like, DNA directed polymerase zeta catalytic subunit	0.10	0.28
MGMT	DR[d]		O-6-methylguanine-DNA methyltransferase	0.09	0.30
ATRIP	Others[k]		ATR interacting protein	0.09	0.32
TREX1	Others[k]	*AGS1, CRV, DRN3, HERNS*	three prime repair exonuclease 1	0.09	0.32
TP53BP1	HDR[f]		tumor protein p53 binding protein 1	0.08	0.27
RAD51	HDR[f]	*FANCR*	RAD51 recombinase	0.08	0.27
POLL	NHEJ[h]		polymerase (DNA directed), lambda	0.07	0.31

a. Frequency of samples with DDR gene copy number gain (GISTIC score = 1) and amplification (GISTIC score = 2) in TCGA PanCanAtlas cohort;

b. Frequency of samples with DDR gene copy number loss (GISTIC score = -1) and amplification (GISTIC score = -2) in TCGA PanCanAtlas cohort;

c. BER: Base Excision Repair; d. DR: Direct Repair; e. FA: Fanconi Anemia; f. HDR: Homology Dependent Recombination; g. MMR: Mismatch Repair; h. NHEJ: Non-homologous End Joining; i. NER: Nucleotide Excision Repair; j. TLS: Translesion Synthesis; k. Others: Damage Sensor and Others

Table S2. Recurrently amplified or deleted DDR genes in TCGA PanCanAtlas cohort (n = 10,489)

Gene Symbol	Recurrently_Amplified_ in_Cancer_Types	*P*-value_mRNA _Amp_vs_Others[a]	Recurrently_Deleted_ in_Cancer_Types	*P*-value_mRNA_ Del_vs_Others[b]
NBN	UCS, BRCA, PRAD, LIHC, BLCA	2.50×10^{-60}		
SHFM1	ESCA, STAD, UCS	3.20×10^{-43}		
UBE2T	BRCA, LIHC, CHOL	2.17×10^{-20}		
EXO1	BRCA, LIHC, OV, CHOL	6.80×10^{-22}		
PARP1	BRCA, CHOL, LIHC, UCS	1.86×10^{-44}		
PRKDC	UCS, LIHC, BRCA	1.20×10^{-34}		
ATR	LUSC, ESCA, CESC OV, HNSC	1.89×10^{-56}		
TOPBP1	LUSC, CESC	5.67×10^{-41}		
POLB	UCS, BRCA, ESCA, LUSC, BLCA	2.16×10^{-111}		
RAD52	UCS, OV	1.84×10^{-59}		
POLQ	LUSC, CESC	7.33×10^{-32}		
EME1	UCS, BRCA	2.78×10^{-28}		
RBBP8	PAAD, ESCA, STAD	1.08×10^{-41}		
CHEK1			TGCT, UVM	0.027
SHPRH			UVM, DLBC	5.99×10^{-10}
REV3L			DLBC, PRAD, UVM	9.18×10^{-17}

a. *P*-value for mRNA expression comparison between samples with specific DDR gene amplification (GISTIC score = 2) and the other copy numbers (Wilcoxon rank-sum test)

b. *P*-value for mRNA expression comparison between samples with specific DDR gene deletion (GISTIC score = -2) and the other copy numbers (Wilcoxon rank-sum test)

Table S3. DDR gene copy number alteration correlates with mRNA expression across TCGA PanCanAtlas patient samples and GDSC cancer cell lines

Gene Symbol	Patient samples			Cancer Cell lines		
	Number_of_Patients	Spearman Rho	Spearman *P*-value	Number_of_Cell_Lines	Spearman Rho	Spearman *P*-value
ALKBH2	8858	0.29	$1.92×10^{-170}$	255	0.04	0.50
ALKBH3	8847	0.34	$2.75×10^{-232}$	659	0.32	$5.00×10^{-17}$
ATM	8829	0.34	$3.55×10^{-233}$	344	0.33	$6.48×10^{-10}$
ATR	8840	0.45	$<10^{-300}$	763	0.26	$3.14×10^{-13}$
ATRIP	8852	0.48	$<10^{-300}$	869	0.26	$4.49×10^{-15}$
BARD1	8830	0.19	$1.91×10^{-69}$	970	0.04	0.19
BLM	8812	0.25	$6.01×10^{-123}$	192	0.07	0.36
BRCA1	8782	0.31	$4.37×10^{-193}$	724	0.13	$5.95×10^{-4}$
BRIP1	8786	0.27	$1.22×10^{-143}$	874	0.07	0.032
CHEK1	8848	0.33	$7.68×10^{-227}$	568	0.27	$9.21×10^{-11}$
CHEK2	8837	0.31	$1.21×10^{-189}$	815	0.16	$3.52×10^{-6}$
CUL5	8834	0.52	$<10^{-300}$	344	0.51	$3.82×10^{-24}$
EME1	8853	0.30	$2.11×10^{-181}$	822	0.18	$2.12×10^{-7}$
ERCC1	8819	0.42	$<10^{-300}$	588	0.28	$6.30×10^{-12}$
ERCC2	8833	0.36	$1.71×10^{-267}$	591	0.27	$1.87×10^{-11}$
ERCC4	8845	0.37	$1.73×10^{-285}$	663	0.21	$8.61×10^{-8}$
ERCC5	8450	0.59	$<10^{-300}$	125	0.59	$4.80×10^{-13}$
ERCC6	8844	0.37	$1.16×10^{-284}$	233	0.02	0.82
EXO1	8828	0.32	$1.37×10^{-207}$	944	0.16	$9.66×10^{-7}$
FANCC	8821	0.45	$<10^{-300}$	197	0.13	0.068
FANCD2	8826	0.36	$3.43×10^{-263}$	19	-0.01	0.95
FANCI	8846	0.31	$4.83×10^{-193}$	216	0.13	0.066
FANCL	8819	0.29	$1.28×10^{-167}$	950	0.15	$4.45×10^{-6}$
FANCM	8828	0.40	$<10^{-300}$	710	0.24	$1.35×10^{-10}$
GEN1	8848	0.32	$2.50×10^{-213}$	970	0.00	0.96
LIG4	8855	0.41	$<10^{-300}$	218	0.35	$7.79×10^{-8}$
MDC1	8854	0.43	$<10^{-300}$	797	0.15	$1.22×10^{-5}$
MGMT	8769	0.26	$4.15×10^{-133}$	500	0.17	$1.54×10^{-4}$
MLH1	8844	0.51	$<10^{-300}$	893	0.30	$2.30×10^{-20}$

MLH3	8851	0.42	$<10^{-300}$	426	0.31	7.68×10^{-11}
MRE11	8822	0.42	$<10^{-300}$	256	0.44	8.50×10^{-14}
MSH2	8809	0.35	3.01×10^{-250}	951	0.15	4.44×10^{-6}
MSH3	8822	0.48	$<10^{-300}$	413	0.21	2.41×10^{-5}
MSH6	8829	0.38	6.40×10^{-295}	947	0.10	2.86×10^{-3}
MUS81	8852	0.37	1.00×10^{-288}	505	0.33	3.88×10^{-14}
NBN	8817	0.41	$<10^{-300}$	374	0.33	4.30×10^{-11}
PALB2	8846	0.41	$<10^{-300}$	618	0.24	9.42×10^{-10}
PARP1	8841	0.54	$<10^{-300}$	938	0.13	5.31×10^{-5}
PMS1	8840	0.34	1.61×10^{-231}	970	0.06	0.083
POLB	8819	0.51	$<10^{-300}$	336	0.49	1.44×10^{-21}
POLE	8831	0.34	1.92×10^{-232}	555	0.13	1.69×10^{-3}
POLE3	8858	0.51	$<10^{-300}$	450	0.09	0.049
POLL	8857	0.52	$<10^{-300}$	68	0.21	0.085
POLM	8816	0.35	6.48×10^{-251}	627	0.16	7.06×10^{-5}
POLN	8775	0.21	3.11×10^{-91}	716	-0.01	0.80
POLQ	8847	0.37	1.70×10^{-277}	550	0.12	4.21×10^{-3}
RAD50	8832	0.43	$<10^{-300}$	704	0.13	4.26×10^{-4}
RAD51	8850	0.19	1.06×10^{-71}	698	0.13	9.19×10^{-4}
RAD52	8825	0.40	$<10^{-300}$	58	-0.01	0.93
RBBP8	8755	0.36	3.97×10^{-266}	518	0.33	2.74×10^{-14}
REV1	8841	0.27	1.25×10^{-146}	30	0.23	0.22
REV3L	8800	0.32	8.12×10^{-204}	477	0.21	2.47×10^{-6}
RNMT	8845	0.52	$<10^{-300}$	506	0.42	1.67×10^{-22}
SEM1	8814	0.42	$<10^{-300}$	212	0.39	5.90×10^{-9}
SHPRH	8835	0.29	3.79×10^{-169}	739	0.20	5.04×10^{-8}
TDG	8855	0.36	3.24×10^{-275}	118	0.20	0.030
TDP1	8835	0.42	$<10^{-300}$	194	0.39	2.29×10^{-8}
TOP3A	8772	0.47	$<10^{-300}$	639	0.28	7.48×10^{-13}
TOPBP1	8845	0.49	$<10^{-300}$	718	0.15	7.01×10^{-5}
TP53BP1	8835	0.37	2.10×10^{-292}	705	0.26	5.88×10^{-12}
TREX1	8856	0.23	1.61×10^{-103}	870	0.13	8.48×10^{-5}

UBE2T	8853	0.37	2.21×10^{-284}	894	0.16	2.27×10^{-6}
UNG	8856	0.39	$<10^{-300}$	256	0.11	0.087
XPA	8850	0.43	$<10^{-300}$	137	0.25	3.67×10^{-3}
XPC	8855	0.40	$<10^{-300}$	752	-0.10	7.74×10^{-3}
XRCC2	8855	0.30	1.33×10^{-183}	851	0.23	7.89×10^{-12}
XRCC3	8847	0.37	3.73×10^{-288}	92	0.24	0.022
XRCC4	8791	0.33	7.74×10^{-224}	373	0.23	5.11×10^{-6}
XRCC5	8845	0.48	$<10^{-300}$	970	0.15	1.60×10^{-6}

Table S4. DDR gene copy number alteration correlates with the drug response (IC50) of 37 genome-instability targeting drugs in 505 GDSC cancer cell lines

Drug_Name	Drug_Target	Drug_Targeted_Pathway	Gene	Spearman *P*-value	Spearman Rho
681640	WEE1, CHEK1	p53 pathway	*APEX1*	0.0416	0.1128
			APEX2	0.0002	0.2552
			EXO1	0.0024	0.1560
			FANCB	0.0190	0.1584
			FEN1	0.0453	0.1407
			MSH2	0.0482	0.1024
			MSH6	0.0498	0.1018
			PARP1	0.0010	0.1688
			PMS1	0.0447	0.1025
			POLM	0.0225	0.1452
			RAD50	0.0349	0.1264
			RAD52	0.0290	0.4880
			REV3L	0.0378	-0.1528
			UBE2T	0.0236	0.1194
5-Fluorouracil	DNA antimetabolite	Genome integrity	*APEX1*	0.0464	0.1004
			APEX2	0.0189	0.1517
			BARD1	0.0357	0.0976
			CHEK2	0.0449	0.1012
			EME1	0.0410	0.1031
			EXO1	0.0271	0.1040
			FANCL	0.0100	0.1211
			FANCM	0.0088	0.1400
			GEN1	0.0131	0.1153
			MGMT	0.0024	0.1974
			MSH2	0.0197	0.1095
			MSH6	0.0062	0.1286
			NHEJ1	0.0200	0.1081
			PMS1	0.0077	0.1237
			PMS2	0.0235	0.1836
			POLM	0.0078	0.1515

			RAD50	0.0006	0.1859
			SHFM1	0.0458	0.1875
			TDP1	0.0464	0.2048
			XRCC2	0.0010	0.1622
			XRCC6	0.0346	0.0985
AG-014699	PARP1, PARP2	DNA replication	*APEX2*	0.0081	0.1696
			EXO1	0.0383	0.0975
			FANCB	0.0015	0.1923
			FANCM	0.0105	0.1386
AT-7519	CDK9	p53 pathway	*BRIP1*	0.0414	0.1003
			EME1	0.0122	0.1260
			ERCC6	0.0114	0.2212
			FANCI	0.0441	0.1988
			FANCL	0.0187	0.1105
			FANCM	0.0158	0.1289
			MGMT	0.0408	0.1338
			MLH3	0.0499	0.1348
			MSH6	0.0419	0.0957
			NHEJ1	0.0480	0.0919
			PMS1	0.0159	0.1118
			RAD50	0.0018	0.1696
			XRCC2	0.0115	0.1246
AZD7762	CHEK1, CHEK2	DNA replication	*APEX1*	0.0045	0.1523
			APEX2	0.0069	0.1830
			ATM	0.0047	0.2247
			ATR	0.0007	0.1895
			BRCA1	0.0068	0.1566
			BRIP1	0.0377	0.1094
			CHEK1	0.0408	0.1290
			CUL5	0.0092	0.2067
			EME1	0.0035	0.1574
			ERCC6	0.0047	0.2817

			FANCL	0.0033	0.1458
			FANCM	0.0008	0.1926
			MGMT	0.0454	0.1403
			MLH3	0.0418	0.1511
			MSH2	0.0063	0.1359
			MSH6	0.0061	0.1366
			PARP1	0.0267	0.1105
			RAD50	0.0412	0.1172
			TOPBP1	0.0008	0.1940
			XPC	0.0026	0.1689
Bleomycin	DNA damage	Genome integrity	*APEX2*	0.0175	0.1548
			ERCC1	0.0490	0.1184
			EXO1	0.0388	0.0983
			FANCB	0.0032	0.1854
			FANCI	0.0143	0.2444
			FANCM	0.0356	0.1145
			PARP1	0.0270	0.1055
			POLB	0.0236	0.1823
			SHFM1	0.0395	0.1940
			UBE2T	0.0227	0.1111
			XPA	0.0425	-0.2627
			XRCC6	0.0377	0.0981
Bleomycin (50 μM)	DNA damage	Genome integrity	*APEX2*	0.0034	0.1851
			FANCB	0.0137	0.1490
			FANCC	0.0028	-0.3208
			NBN	0.0136	0.1822
			POLB	0.0158	0.1900
			XPA	0.0116	-0.3185
Camptothecin	TOP1	Genome integrity	*APEX1*	0.0082	0.1416
			APEX2	0.0071	0.1824
			ATR	0.0085	0.1469
			BARD1	0.0406	0.1009

			CHEK2	0.0269	0.1171
			EME1	0.0420	0.1098
			ERCC4	0.0179	0.1416
			ERCC6	0.0022	0.3046
			EXO1	0.0006	0.1704
			FANCL	0.0056	0.1374
			FANCM	0.0006	0.1989
			FEN1	0.0272	0.1517
			GEN1	0.0330	0.1050
			LIG4	0.0194	0.2396
			MDC1	0.0360	0.1157
			MGMT	0.0026	0.2097
			MSH2	0.0098	0.1285
			MSH6	0.0150	0.1213
			NBN	0.0090	0.2071
			NHEJ1	0.0285	0.1079
			PALB2	0.0325	0.1339
			PARP1	0.0005	0.1729
			PMS1	0.0311	0.1062
			RAD50	0.0253	0.1285
			REV3L	0.0171	-0.1689
			SLX1A	0.0430	0.1321
			TDG	0.0377	0.3143
			TOPBP1	0.0146	0.1411
			UBE2T	0.0077	0.1349
			XPC	0.0275	0.1239
CGP-082996	CDK4	p53 pathway	*FANCA*	0.0358	-0.1651
			FANCC	0.0218	-0.4396
			LIG4	0.0171	0.3620
			PMS2	0.0389	0.2794
			SHFM1	0.0335	0.3710
			XPA	0.0223	-0.4956

CGP-60474	CDK1,CDK2,CDK5,CDK7,CDK9	p53 pathway	*APEX2*	0.0151	0.2487
			ERCC6	0.0227	0.3354
			FANCC	0.0430	-0.3922
			FANCM	0.0160	0.2040
			PMS1	0.0486	0.1437
Cisplatin	DNA crosslinker	Genome integrity	*NBN*	0.0016	0.2498
			REV3L	0.0381	-0.1472
			XPA	0.0363	-0.2856
CP466722	ATM	DNA replication	*APEX1*	0.0113	0.1270
			ATR	0.0217	0.1198
			BRCA2	0.0054	0.1425
			CHEK2	0.0290	0.1096
			EME1	0.0272	0.1107
			ERCC4	0.0493	0.1088
			ERCC6	0.0097	0.2260
			FANCL	0.0159	0.1129
			FANCM	0.0087	0.1397
			GEN1	0.0399	0.0951
			MLH3	0.0243	0.1543
			PMS1	0.0038	0.1337
			PMS2	0.0292	0.1764
			POLQ	0.0112	0.1583
			RAD50	0.0321	0.1163
			SHFM1	0.0267	0.2076
			TDP1	0.0312	0.2224
			TOP3A	0.0096	0.1481
			TOPBP1	0.0085	0.1410
			XPC	0.0193	0.1227
			XRCC2	0.0383	0.1019
Cytarabine	DNA synthesis	Genome integrity	*ATM*	0.0244	0.1808
			ATR	0.0436	0.1132
			CUL5	0.0243	0.1802

			ERCC6	0.0318	0.2170
			EXO1	0.0115	0.1261
			PARP1	0.0378	0.1038
			REV3L	0.0113	-0.1797
			TOPBP1	0.0211	0.1338
Doxorubicin	DNA intercalating	Genome integrity	*BRIP1*	0.0235	0.1123
			ERCC1	0.0323	0.1286
			ERCC2	0.0393	0.1235
			ERCC5	0.0218	0.2820
			ERCC6	0.0134	0.2173
			FANCC	0.0157	-0.2676
			FANCI	0.0034	0.2891
			FANCM	0.0253	0.1214
			POLB	0.0115	0.2024
			XPA	0.0009	-0.4160
			XRCC2	0.0490	0.0978
Etoposide	TOP2	Genome integrity	*ERCC4*	0.0199	0.1305
			ERCC5	0.0016	0.3759
			ERCC6	0.0047	0.2475
			FANCC	0.0355	-0.2340
			FANCI	0.0247	0.2223
			FANCM	0.0016	0.1700
			LIG4	0.0305	0.2074
			MGMT	0.0162	0.1570
			RAD52	0.0167	0.4738
			REV3L	0.0014	-0.2122
			SHPRH	0.0128	-0.1349
			XPA	0.0061	-0.3499
Gemcitabine	DNA replication	Genome integrity	*APEX2*	0.0287	0.1430
			BRIP1	0.0424	0.1009
			ERCC5	0.0083	0.3226
			ERCC6	0.0152	0.2150

			FANCB	0.0319	0.1358
			FANCM	0.0344	0.1153
			LIG4	0.0447	0.1945
			MGMT	0.0148	0.1599
			REV3L	0.0395	-0.1380
JNJ-26854165	MDM2	cell cycle	*APEX2*	0.0031	0.1897
			ATR	0.0415	-0.1074
			FANCA	0.0316	-0.1087
			POLQ	0.0314	-0.1358
			RAD50	0.0370	-0.1146
			REV3L	0.0190	-0.1573
			TOP3A	0.0106	-0.1483
			XPC	0.0172	-0.1260
			XRCC4	0.0248	-0.1683
			XRCC6	0.0025	-0.1412
KIN001-270	CDK9	p53 pathway	*APEX2*	0.0312	0.1385
			BARD1	0.0411	0.0946
			EME1	0.0214	0.1154
			EXO1	0.0168	0.1120
			FANCL	0.0126	0.1168
			FANCM	0.0001	0.2021
			GEN1	0.0158	0.1117
			MGMT	0.0112	0.1646
			MSH2	0.0094	0.1214
			MSH6	0.0042	0.1338
			NHEJ1	0.0324	0.0990
			PMS1	0.0023	0.1410
			PMS2	0.0254	0.1812
			POLM	0.0028	0.1699
			RAD50	0.0004	0.1913
			REV3L	0.0212	-0.1526
KU-55933	ATM	DNA replication	*APEX2*	0.0010	0.2193

			ATR	0.0181	0.1295
			BARD1	0.0425	0.0993
			FANCM	0.0231	0.1301
			GEN1	0.0259	0.1090
			NHEJ1	0.0271	0.1081
			PALB2	0.0198	-0.1467
			PMS1	0.0031	0.1444
			POLM	0.0102	0.1565
			POLQ	0.0266	0.1478
			RAD50	0.0208	0.1330
			RAD52	0.0054	0.5000
			TOPBP1	0.0141	0.1391
			XPC	0.0083	0.1455
			XRCC5	0.0470	0.0972
Methotrexate	Dihydrofolate reductase (DHFR)	Genome integrity	*APEX1*	0.0035	0.1564
			ATR	0.0129	0.1393
			EXO1	0.0367	0.1044
			FANCM	0.0066	0.1573
			MDC1	0.0385	0.1143
			MSH2	0.0333	0.1062
			MSH6	0.0233	0.1133
			PARP1	0.0291	0.1090
			POLM	0.0129	0.1508
			RAD50	0.0002	0.2115
			RAD52	0.0000	0.5000
			TOPBP1	0.0344	0.1228
			XPC	0.0134	0.1392
Mitomycin C	DNA crosslinker	Genome integrity	*BRIP1*	0.0491	0.0976
			FANCB	0.0100	0.1617
			FANCM	0.0437	0.1096
			MLH1	0.0425	0.0989
			NHEJ1	0.0448	0.0942

			POLB	0.0132	0.1987
			REV3L	0.0317	-0.1439
			XRCC2	0.0290	0.1084
NSC-207895	MDMX	cell cycle	*ATR*	0.0276	0.1155
			BARD1	0.0093	0.1207
			BRIP1	0.0369	0.1027
			EME1	0.0080	0.1336
			ERCC4	0.0277	0.1225
			ERCC5	0.0232	0.2793
			ERCC6	0.0133	0.2183
			EXO1	0.0403	0.0965
			FANCA	0.0321	0.1079
			FANCL	0.0087	0.1234
			FANCM	0.0020	0.1646
			GEN1	0.0246	0.1044
			MGMT	0.0070	0.1756
			MSH2	0.0039	0.1352
			MSH6	0.0042	0.1344
			NHEJ1	0.0191	0.1089
			PMS1	0.0077	0.1237
			POLM	0.0160	0.1376
			RAD50	0.0015	0.1719
			REV3L	0.0238	-0.1510
			TOPBP1	0.0091	0.1405
			XRCC2	0.0370	0.1028
			XRCC4	0.0416	0.1524
			XRCC5	0.0248	0.1043
NU-7441	DNAPK	DNA replication	*APEX1*	0.0326	0.1134
			APEX2	0.0080	0.1771
			ATR	0.0024	0.1665
			BARD1	0.0192	0.1147
			BRCA1	0.0123	0.1422

			BRIP1	0.0227	0.1182
			EME1	0.0019	0.1644
			ERCC6	0.0154	0.2370
			EXO1	0.0032	0.1458
			FANCL	0.0025	0.1496
			FANCM	0.0024	0.1737
			GEN1	0.0124	0.1225
			MDC1	0.0271	0.1211
			MSH2	0.0014	0.1582
			MSH6	0.0015	0.1577
			NHEJ1	0.0060	0.1346
			PARP1	0.0037	0.1439
			PMS1	0.0026	0.1472
			POLM	0.0073	0.1637
			POLQ	0.0048	0.1883
			RAD50	0.0004	0.2044
			RAD52	0.0098	0.5000
			TOPBP1	0.0019	0.1757
			UBE2T	0.0165	0.1214
			XPC	0.0022	0.1687
			XRCC5	0.0220	0.1123
Nutlin-3a (-)	MDM2	cell cycle	*APEX1*	0.0001	0.2066
			ATR	0.0009	0.1814
			BLM	0.0405	0.2254
			CHEK2	0.0055	0.1463
			EME1	0.0490	0.1043
			ERCC6	0.0059	0.2657
			EXO1	0.0183	0.1166
			FANCI	0.0042	0.2929
			FANCL	0.0003	0.1770
			FANCM	0.0001	0.2296
			FEN1	0.0497	0.1328

			GEN1	0.0044	0.1387
			MDC1	0.0008	0.1819
			MLH3	0.0061	0.1988
			MSH2	0.0003	0.1762
			MSH6	0.0001	0.1876
			MUS81	0.0467	0.1328
			PARP1	0.0105	0.1266
			PMS1	0.0035	0.1422
			POLE3	0.0104	0.1875
			POLE3	0.0104	0.1875
			POLM	0.0052	0.1701
			RAD50	0.0280	0.1262
			RNMT	0.0037	0.1936
			SHPRH	0.0470	0.1131
			TDG	0.0212	0.3354
			TOPBP1	0.0152	0.1375
			XPC	0.0042	0.1578
			XRCC6	0.0474	0.0973
Olaparib	PARP1, PARP2	DNA replication	*APEX1*	0.0173	0.1275
			ATR	0.0030	0.1652
			ATR	0.0191	0.1240
			BARD1	0.0041	0.1410
			BLM	0.0051	0.2913
			BRCA1	0.0328	0.1237
			BRIP1	0.0305	0.1138
			EME1	0.0437	0.1090
			ERCC6	0.0343	0.2130
			EXO1	0.0016	0.1569
			FANCB	0.0202	0.1511
			FANCI	0.0000	0.3971
			FANCI	0.0424	0.2098
			FANCL	0.0006	0.1704

			FANCM	0.0005	0.2015
			FANCM	0.0028	0.1608
			GEN1	0.0005	0.1704
			MSH2	0.0010	0.1638
			MSH6	0.0005	0.1732
			NBN	0.0046	0.2242
			NHEJ1	0.0370	0.0972
			NHEJ1	0.0035	0.1434
			PARP1	0.0032	0.1467
			PMS1	0.0011	0.1599
			POLM	0.0482	0.1138
			RAD50	0.0205	0.1328
			REV3L	0.0014	-0.2134
			TOPBP1	0.0350	0.1220
			TOPBP1	0.0384	0.1127
			XPC	0.0166	0.1345
			XRCC5	0.0064	0.1339
PD-0332991	CDK4, CDK6	p53 pathway	*APEX1*	0.0352	0.1148
			APEX2	0.0271	0.1507
			FANCM	0.0114	0.1491
			MDC1	0.0408	0.1142
PHA-793887	CDK-pan	p53 pathway	*APEX1*	0.0431	0.1017
			APEX2	0.0186	0.1518
			EME1	0.0274	0.1107
			ERCC6	0.0321	0.1888
			FANCI	0.0140	0.2404
			FANCL	0.0061	0.1283
			FANCM	0.0163	0.1281
			MGMT	0.0109	0.1659
			MSH6	0.0449	0.0942
			NHEJ1	0.0462	0.0924
			PMS1	0.0231	0.1052

			POLM	0.0492	0.1124
			RAD50	0.0007	0.1825
			XRCC2	0.0005	0.1706
RO-3306	CDK1	RO-3306	*APEX2*	0.0137	0.1649
			MRE11A	0.0379	0.1914
Roscovitine	CDK family	Roscovitine	*FANCM*	0.0146	0.2074
			REV3L	0.0195	-0.2473
SN-38	TOP1	Genome integrity	*APEX2*	0.0464	0.1269
			EXO1	0.0178	0.1107
			FANCB	0.0382	0.1257
			FANCI	0.0158	0.2374
			FANCM	0.0018	0.1667
			PARP1	0.0157	0.1131
			REV3L	0.0091	-0.1728
Talazoparib	PARP1, PARP2	DNA replication	*FANCB*	0.0122	0.1537
			REV3L	0.0261	-0.1486
Temozolomide	DNA alkylating agent	Genome integrity	*APEX1*	0.0179	0.1189
			APEX2	0.0026	0.1929
			ATR	0.0386	0.1094
			BARD1	0.0156	0.1125
			BLM	0.0454	0.2103
			EXO1	0.0467	0.0938
			FANCI	0.0275	0.2162
			FANCL	0.0461	0.0939
			FANCM	0.0005	0.1877
			GEN1	0.0034	0.1359
			MRE11A	0.0237	0.1976
			MSH2	0.0124	0.1176
			MSH6	0.0169	0.1124
			NHEJ1	0.0111	0.1180
			PARP1	0.0321	0.1013
			PMS1	0.0028	0.1386

			RAD50	0.0016	0.1721
			REV3L	0.0103	-0.1722
			XRCC5	0.0362	0.0975
THZ-2-102-1	CDK7	p53 pathway	*APEX1*	0.0028	0.1505
			BRIP1	0.0227	0.1123
			EME1	0.0109	0.1285
			ERCC6	0.0083	0.2323
			FANCI	0.0268	0.2182
			FANCL	0.0172	0.1122
			FANCM	0.0000	0.2417
			MLH3	0.0109	0.1750
			MSH3	0.0148	0.1752
			MSH6	0.0474	0.0935
			PMS1	0.0311	0.1003
			PMS2	0.0032	0.2381
			POLM	0.0051	0.1601
			POLQ	0.0488	0.1238
			PRKDC	0.0080	0.2115
			RAD50	0.0001	0.2080
			REV3L	0.0355	-0.1406
			SHFM1	0.0077	0.2507
			TOPBP1	0.0096	0.1394
			XRCC2	0.0088	0.1292
			XRCC4	0.0118	0.1890
THZ-2-49	CDK9	p53 pathway	*FANCM*	0.0140	0.1313
			MLH3	0.0238	0.1549
			PRKDC	0.0369	0.1673
Veliparib	PARP1, PARP2	DNA replication	*APEX1*	0.0000	0.2452
			APEX2	0.0024	0.2049
			ATR	0.0012	0.1801
			BARD1	0.0012	0.1589
			BRCA1	0.0234	0.1315

			BRIP1	0.0330	0.1122
			ERCC6	0.0060	0.2744
			EXO1	0.0001	0.1992
			FANCB	0.0024	0.1971
			FANCL	0.0003	0.1805
			FANCM	0.0000	0.2595
			GEN1	0.0007	0.1664
			MDC1	0.0356	0.1159
			MSH2	0.0001	0.1892
			MSH6	0.0002	0.1875
			NBN	0.0070	0.2136
			NHEJ1	0.0019	0.1523
			PARP1	0.0001	0.1926
			PMS1	0.0002	0.1842
			POLE3	0.0105	0.1903
			POLE3	0.0105	0.1903
			POLM	0.0283	0.1334
			POLQ	0.0087	0.1765
			RAD50	0.0007	0.1933
			TDG	0.0335	0.3213
			TOPBP1	0.0046	0.1633
			UBE2T	0.0187	0.1192
			XPC	0.0080	0.1488
			XRCC5	0.0016	0.1549
